# Ontario public safety personnel experiences of workplace mental wellness supports

**DOI:** 10.1371/journal.pmen.0000558

**Published:** 2026-06-08

**Authors:** Megan Edgelow, Yeganeh Mousavi, Alexa Paemurd, Nazmul Islam, Larissa Oliveira, Grace Moffat

**Affiliations:** 1 School of Rehabilitation Therapy, Queen’s University, Kingston, Ontario, Canada; 2 Department of Psychology, Queen’s University, Kingston, Ontario, Canada; 3 School of Computing, Queen’s University, Kingston, Ontario, Canada; 4 Department of Pathology and Molecular Medicine, Queen’s University, Kingston‌‌, Ontario, Canada; 5 Department of Politics and Public Administration, Toronto Metropolitan University, Toronto, Ontario, Canada; National University of Singapore, SINGAPORE

## Abstract

In Canada, the rates and costs of psychological injury for public safety personnel (PSP) have been rising for the past decade. Public safety organizations often support the mental wellness of their employees with individually focused interventions, neglecting the impact of organizational factors. This study aimed to understand the mental wellness and organizational experiences of PSP in the province of Ontario, Canada, including border services officers, communicators, correctional workers, firefighters, paramedics, and police officers. This cross-sectional convergent mixed methods study included an online survey (June-December 2024) and follow-up virtual semi-structured interviews (January-February 2025). Quantitative and qualitative data were analyzed using descriptive statistics and framework analysis, respectively. The 644 survey participants showed a low to moderate risk of serious mental illness, an above average risk of work-related stress, and a low to average level of work engagement on standardized measures. Border services officers and correctional workers showed a pattern of higher work stress and mental distress, with lower work engagement, while firefighters and police displayed the opposite pattern. Available employer-funded supports included internal peer support programs (83%), critical incident debriefing (62%), external mental health supports (61%), and extended health benefits (77%). The 15 follow up interview participants identified low staffing, low control at work, and conflict between supervisors and coworkers as chronic work-related stressors. Overall, survey and interview participants had low to moderate intentions for the use of internal resources and supports and conflicting views of their trustworthiness and effectiveness, and endorsed the need for more tailored internal mental wellness supports as well as a preference for the use of extended health benefits. This study is the first to capture Ontario public safety employer efforts to support employee mental wellness and employee experiences of these efforts, revealing mixed perceptions of quality. Findings can be used to consider the most impactful use of organizational resources.

## Introduction

### Background‌‌

Public safety personnel (PSP) in Canada include border services officers, communicators, correctional workers, firefighters, paramedics, and police officers, among others [1]. These individuals serve across municipal, provincial, and federal jurisdictions and routinely encounter potentially psychologically traumatic events (PPTEs), including direct or indirect exposure to death, violence, suicide, and threats to personal safety [2]. Such repeated exposure places them at elevated risk for a range of psychological injuries, including burnout, diagnoses of posttraumatic stress disorder (PTSD), depression, anxiety disorders, and substance use disorders, and suicidal ideation [3,4]. Research suggests that moral injury can also affect PSP when they are exposed to situations that conflict with their core ethical values, such as being unable to prevent harm or being required to follow protocols they perceive as morally wrong [5].

The prevalence of work-related psychological injury among PSP is thought to be significantly higher than in the general population. A large-scale national study involving 5,813 Canadian PSP found that 44.5 percent of the participants screened positive for one or more mental disorders [2]. Psychological injuries not only affect individual well-being but also lead to reduced job performance, time off work, early retirement, and significant system-wide costs, including increased long-term workers’ compensation and disability claims [6,7]. A recent study of Ontario workers’ compensation data revealed 11,306 approved mental stress injury program claims made by PSP between 2014 and 2023, including communicators, correctional workers, firefighters, paramedics, and police [8]. The vast majority of these claims resulted in time lost from work (93%), with a median claim length of more than one year, and only a third of claimants successfully returning to work, representing a large human and financial burden for Ontario public safety employers, tax payers, PSP, their families, and communities [8].

Operational, organizational, and personal factors intersect to influence PSP mental health, highlighting the complexity of workplace exposures [9]. Although workplace mental health programs have been widely implemented across public safety sectors, evidence of their effectiveness remains inconsistent [10]. Many initiatives fail to address organizational contributors to distress, such as toxic culture, poor leadership, or structural barriers to accessing care [3,11]. Furthermore, PSP are not a uniform group; for example, correctional workers face different stressors than paramedics or border services officers. These occupational differences have significant implications for intervention design, stigma levels, and return to work (RTW) outcomes [4,7]. Despite this, many workplace interventions are designed for general mental health support, rather than being tailored to the specific needs of each PSP subgroup. This lack of customization undermines program relevance and effectiveness, as interventions that do not reflect the distinct realities of each occupation are often dismissed by PSP or poorly integrated into practice [12].

Exposure to critical incidents remains a common operational challenge among paramedics, police, and firefighters, elevating their risk of trauma-related disorders [2,13]. Moral conflict and ambiguous public roles are evident among correctional workers, communicators, and border services officers, often producing ethical strain and feelings of helplessness [4,14]. Stigma and fear of judgment remain persistent barriers across all sectors, potentially limiting help-seeking and delaying work reintegration [3,4]. PSP working in rural areas may report more psychological distress than those in urban or mixed settings, and factors such as supervisor support, peer cohesion, and workplace culture have been found to significantly influence mental health outcomes [6]. Ultimately, while each PSP career group operates under unique pressures, the cumulative weight of trauma exposure, resource limitations, and negative workplace culture can create converging barriers to recovery from psychological injuries and RTW. For psychologically injured PSP, RTW outcomes are often shaped by the presence of organizational stressors, workplace stigma, and the availability of peer and supervisory support which can differ widely across PSP populations and workplace contexts. Research indicates that systemic issues in workers’ compensation processes and PSP workplaces, including claim and treatment delays, stigma, and inconsistent accommodations, continue to affect PSP recovery and work reintegration [6,8,15]. The RTW processes for PSP in Ontario have been reported by PSP as negative, with low satisfaction scores and common experiences of psychologically unsafe environments [15,16]. Many PSP described stigma, inadequate workplace accommodations, and inconsistent employer support as major barriers to successful reintegration [15].

In response to the high rates of psychological injury among Canada’s PSP, numerous mental health programs have been introduced across federal, provincial, and municipal levels. These include both broad wellness initiatives and structured interventions aimed at mitigating the mental health effects of operational and organizational stress. These interventions can be categorized into two main types: general wellness programs focused on stress education, mindfulness, and physical health; and named, replicable programs such as Critical Incident Stress Debriefing (CISD), Road to Mental Readiness (R2MR), and Mindfulness-Based Stress Reduction (MBSR) [17]. Despite the widespread implementation of these programs, evidence supporting long-term effectiveness remains limited, and substantial gaps persist in program evaluation, access, and cultural fit across different PSP groups [10,17].

A recent scoping review revealed that across all PSP workplace mental health intervention types, there was a dominant focus on individual coping strategies, often at the expense of addressing the structural and organizational sources of distress. PSP across sectors described most workplace mental health initiatives as checkbox exercises, insufficient in both delivery and relevance [17]. Many interventions, especially online modules, were perceived as generic, poorly tailored to PSP realities, and devoid of sustained engagement. The result, according to participants, was minimal impact on resilience or workplace safety, especially in psychologically unsafe environments shaped by stigma and poor leadership. Similar studies echo these concerns, noting that few programs demonstrated consistent clinical outcomes, and even those with short-term benefits lacked longitudinal evidence [10]. Importantly, only a limited number of programs explicitly targeted leadership, despite strong evidence that supervisor involvement can reduce stigma and improve mental health outcomes [17–19]. A recent study of workplace programs to reduce work disability for Alberta firefighters, paramedics, and police revealed that programs were frequently viewed as being under development, identifying the need to streamline processes and improve programs and resources, with a need for further research to understand effective program implementation [20].

### Objectives

This study explored the mental wellness and organizational experiences of Ontario PSP, including border services officers, communicators, correctional workers, firefighters, paramedics, and police. By highlighting key differences across public safety occupations and examining organizational and policy-level influences on mental wellness support, this study aimed to identify positive factors, as well as concerning factors and gaps, and propose directions for mental wellness support in public safety organizations.

## Materials and methods

### Ethics statement

The research received ethical review and approval from the Health Sciences Research Ethics Board (HSREB) at Queen’s University (REH-878–23) and participants consented in writing before beginning the survey and consented verbally before beginning an interview.

### Study design

This cross-sectional convergent mixed methods study included an online survey (June-December 2024) and follow-up virtual semi-structured interviews (January-February 2025).

#### Survey.

The survey was hosted on the virtual platform RedCap, as web-based surveys have been found to be more consistent than other methods of surveying [21]. Using an anonymous web-based survey may capture more honest opinions as opposed to just using in person interviews, where participants may be inclined to provide socially acceptable answers [22,23]. The survey began by collecting demographic information (e.g., profession, gender identity, age, years of experience) and proceeded with three self-report standardized measures: the Health & Safety Executive Management Standards Indicator Tool (HSE-MS IT), the Kessler Psychological Distress Scale (K6), and the Utrecht Work Engagement Scale (UWES-9). The survey concluded with check box and open-text questions about mental wellness programs, resources, and supports available within the employment environment.

The three standardized measures are all self-report instruments that use Likert scaling and have been previously validated for research on work-related stress, psychological distress, and work engagement, respectively [24–26]. The standardized measures were also chosen for their relative brevity in comparison to other tools measuring similar concepts. The survey was designed to limit survey length-related dropout rates by reducing response demands once the standardized measures, check box, and open-text questions were combined. The HSE-MS IT is commonly used to assess work-related stress risks at an organizational level [24]. It has 35 structured items rated on a scale of 1–5, where 1 indicates a poor score and 5 indicates a desirable score, across seven domains: change, control, demands, manager’s support, peer support, relationships, and role. The K6 screens for the presence of mental illness and includes six items that measure the frequency of how often the participant felt: nervous; hopeless; restless or fidgety; so depressed that nothing could cheer you up; that everything was an effort; and worthless [25]. Responses are rated on a five-point scale ranging from “none of the time” (0) to “all of the time” (4), with higher cumulative scores indicating greater levels of distress. The UWES-9 is a nine-item measure used to assess work engagement across three subscales: absorption, dedication, and vigor [26]. Each item refers to the frequency of workplace experiences and is rated on a seven-point scale ranging from “never” (0) to “always” (6), with higher scores indicating greater levels of work engagement.

The survey concluded with questions about mental wellness programs, resources, and supports available within the employment environment, using check box and open-text responses. Resource and support categories included peer support, debriefing, internal and external mental health services, training and resources, extended health benefits, flexibility in the workplace, and other resources and supports. These broad categories were chosen to reflect mental wellness resources and supports that have appeared in organizational research and are known to exist within the Ontario public safety context [3,17,20]. Participants were asked to first indicate the availability of programs, resources, and supports through check box questions; those with personal experience or knowledge of these items were prompted with open-text questions to gather feedback, concerns, and intentions for future use.

#### Interviews.

Zoom, a virtual meeting platform, was used to facilitate virtual follow-up interviews. The semi-structured interview script was developed using key topics from the survey, including the domains of the HSE-MS IT (change, control, demands, manager’s support, peer support, relationships, and role). The interview script also prompted participants to report on programs, resources, and supports available within their employment environments, and mirrored the survey’s check box and open-text categories.

### Setting and participants

The survey was open to Ontario PSP and was targeted to six main occupational groups: border services officers, communicators, correctional workers, firefighters, paramedics, and police. A web link to the survey was shared via email to key stakeholder groups and public safety employers and also shared via social media platforms between June 1-December 31, 2024. Since the survey link was accessible over multiple platforms, there is no way to accurately estimate the number of individuals who were invited to participate in the survey.

At the conclusion of the survey, participants were invited to provide their contact information in a separate survey if they were willing to be contacted for a follow up interview. Three spots were allocated per key profession (border services officers, communicators, correctional workers, firefighters, paramedics, and police), for a total of 18 potential interviews. In January 2025, interested participants were invited via email to sign up for follow up interviews via the online appointment booking platform Calendly, with interviews operating on a first come, first served basis for the available spots per profession. Interviews took place in January and February 2025.

### Data analysis

Quantitative and qualitative data analysis was performed using Microsoft Excel, with R Studio (version 2025.05.1) supporting deeper quantitative analysis of survey data. Frequency counts and descriptive analyses were initially performed for quantitative data collected from the survey and follow up interviews. Additionally, to analyze the survey data from the three standardized measures, we first assessed normal distribution using the Shapiro–Wilk test. Most subscales were non-normally distributed, given the nature of our dataset and the unequal group sizes. Thus, the non-parametric Kruskal–Wallis test was used to assess whether distributions differed significantly between groups, followed by pairwise comparisons using the post-hoc Dunn test with p-values adjusted by the Benjamini–Hochberg (BH) method to control the false discovery rate. Effect sizes for Kruskal–Wallis tests were calculated using epsilon-squared (ε²), with values of 0.01, 0.06, and 0.14 considered small, moderate, and large effects. To investigate whether differences on standardized measures across occupation were affected by demographic variables such as gender, race, and sexuality, we performed a multivariable linear regression model, which included occupation, gender, race, and sexuality as predictors. Due the small sample size for many of the subcategories of race and sexuality, we transformed the data into a binary classification (straight and LGBTQ+ for sexuality, and white and non-white for race).

Qualitative framework analysis was conducted to identify the major themes emerging from open-text survey responses and the semi-structured interviews. Employing an interpretive approach for open-text survey and interview data, framework analysis provided a systematic and comprehensive analytic method to analyze qualitative data [27]. The qualitative analysis was guided by Ritchie and Spencer’s five stages of framework analysis: familiarizing, identifying a thematic framework, indexing, charting, mapping, and interpreting [27]. In the context of framework analysis, saturation refers to the point during the mapping and interpretation stage where reviewing additional data does not produce any new themes, codes, or variations within the existing framework categories [27]. While mapping the data into the framework matrix, saturation was reached when subsequent data points only filled in existing cells, rather than requiring new codes or changing the structural understanding of the themes.

The lead author and researcher recognizes that her personal background as a white woman with graduate level education influences her perspective. Although she has experience working clinically with military members, veterans, and public safety personnel and understands occupational risk factors, she is not a member of these communities. Additionally, although many study participants share demographic and educational similarities with her, experiences are not universal. The lead author’s experience as an occupational therapist means she entered this study with a predisposition toward focusing on structural issues and prevention of mental stress injuries over individual interventions. To ensure her subjectivity did not overshadow the participants’ experiences, she actively discussed her interpretations with her co-authors, who have a variety of racial and professional backgrounds. She also consulted with public safety personnel and researchers in the field on key interpretations of the data and presented the results at several conferences before moving to publication, in order to receive peer feedback. She recognizes that she is not a neutral observer, but rather a participant in the social construction of knowledge.

## Results

### Survey

Survey participants who met the inclusion criteria of being an Ontario PSP and who completed at least the demographic information and the first standardized measure (HSE-MS IT) of the survey were included in the analysis of this study. Six hundred forty-four survey participants (N = 644) were included, while 317 participants were excluded for not providing adequate responses or meeting the inclusion criteria. All included participants completed the demographic data and the HSE-MS IT measure; each subsequent standardized measure and the open-text questions experienced a small reduction in participant numbers due to drop-out effects, and rates of participation in each aspect of the survey can be seen in Tables 2, 4, 6 and 8.

#### Demographics.

Table 1 outlines the demographic information for the respondents who met the inclusion criteria of the survey (N = 644). 52% of respondents (n = 337) reported their gender identity as a man, while 47% (n = 300) identified as a woman. Most of the sample (86%, n = 551) identified their racial identity as white and their sexual orientation as straight (89%, n = 571). For more information on participants who identified with different gender or racial identities, or sexual orientation, or preferred not to disclose this information, see Table 1. The sample included a variety of PSP occupations: border services officers (23%, n = 145), communicators (3%, n = 22), correctional workers (24%, n = 152), firefighters (14%, n = 88), paramedics (21%, n = 133), police (14%, n = 88), and 2% (n = 16) selected the “other” category. In the “other” category, 4 participants were federal emergency managers, and 8 participants were provincial health and safety inspectors. Notably, most correctional workers were provincial employees (97%, n = 147) and most police were municipal employees (85%, n = 75), with low response rates from correctional workers in federal prisons (1%, n = 2) and federal police (6%, n = 5). Firefighters were also mostly municipal workers (73%, n = 64), with other respondents working in wildland firefighting both provincially and federally. Other career groups responded as expected within typical employment contexts, with 99% (n = 144) of border services officers employed federally, and communicators and paramedics employed across jurisdictions. The lowest response rate was for communicators at 3% of respondents (n = 22); this occupational group can be difficult to recruit for research purposes as they are often employed within other services and lack a provincial association or distinct union.

**Table 1:** Survey demographics.

		Occupations							
		Border Services Officers	Communicators	Correctional Workers	Firefighters	Paramedics	Police	Other	Total (N)
**Total Respondents**		145	22	152	88	133	88	16	644
**Gender Identity**	**Woman**	52	15	69	32	76	48	8	300
	**Man**	92	7	80	54	56	40	8	337
	**Other**	1	0	3	2	1	0	0	7
**Sexual Orientation**	**Bisexual**	5	0	2	4	5	4	0	20
	**Gay**	2	0	2	0	0	0	1	5
	**Lesbian**	1	2	4	1	7	0	1	16
	**Straight**	129	20	137	75	114	83	13	571
	**Prefer not to Answer**	5	0	4	2	2	1	0	14
	**Other**	3	0	3	6	5	0	1	18
**Racial Identity**	**Asian**	4	3	6	3	4	1	0	21
	**Black**	2	1	3	3	0	11	1	21
	**Indigenous**	4	0	7	4	3	1	0	19
	**Latin American**	2	0	1	1	3	0	0	7
	**White**	127	18	126	76	119	72	13	551
	**Prefer Not to Say**	4	0	5	0	4	3	1	17
	**Other**	2	0	4	2	0	0	1	9
**Average Age in Years** **(SD)**		42.3 (8.8)	35.6 (9.5)	42.8 (10.4)	41.0 (12.2)	40.4 (10.7)	38.3 (9.5)	49.3 (9.2)	41.2 (10.4)
**Average Years of Work Experience (SD)**		15.3 (8.8)	7.7 (6.3)	15.1 (9.9)	13.2 (10.2)	14.8 (10.0)	11.9 (9.6)	13.8 (8.4)	14.1 (9.7)
**Jurisdiction**	**Federal**	144	3	2	8	5	5	4	171
	**Municipal**	0	7	3	64	95	75	0	244
	**Provincial**	0	12	147	14	27	7	12	219
	**Other**	1	0	0	2	6	1	0	10

#### Standardized measures.

Table 2 displays the HSE-MS IT subscales (means and medians) and their distribution across all PSP groups. First, we performed a Shapiro-Wilk test and demonstrated that most subscales did not present a normal distribution, with W varying from 0.93 to 0.99 (p < 0.05). We then performed a Kruskal–Wallis test to assess whether there was a different distribution of scores across occupations. Significant differences were observed for all HSE-IT domains (p < 0.001), with effect sizes ranging from moderate to large (ε² = 0.09–0.27) (Table 3). We then investigated which occupations differed from each other within each subscale by doing a post-hoc pairwise comparisons using Dunn’s test, with p-values adjusted using the BH method (all p-values are displayed in S1 Table).

**Table 2:** Mean and median HSE-MS IT subscale scores.

	Occupations							
	Border Services Officers	Communicators	Correctional Workers	Firefighters	Paramedics	Police	Other	Total
**Respondents (N)**	145	22	152	88	133	88	16	644
**Change** **(Mean ± SD)**	1.8 ± 0.7	2.2 ± 1.0	1.8 ± 0.7	3.1 ± 1.0	2.3 ± 0.9	3.1 ± 0.9	2.2 ± 0.9	2.3 ± 1.0
**Change (Median [Q1-Q3])**	1.6 [1.3–2.3]	2.6 [1.0–3.2]	1.6 [1.3–2.3]	3.3 [2.3–4.0]	2.3 [1.6–3.0]	3.3 [2.6–3.6]	2.1 [1.3–3.0]	2.0 [1.3–3.0]
**Control** **(Mean ± SD)**	2.9 ± 0.6	2.6 ± 0.8	2.7 ± 0.7	3.3 ± 0.7	2.6 ± 0.9	3.5 ± 0.6	3.4 ± 0.6	3.0 ± 0.8
**Control (Median [Q1-Q3])**	3.0 [2.4–3.4]	2.5 [2.0–3.5]	2.6 [2.2–3.2]	3.3 [2.9–3.8]	2.6 [2.0–3.2]	3.8 [3.2–4.0]	3.3 [3.1–3.8]	3.0 [2.4–3.6]
**Demands** **(Mean ± SD)**	3.1 ± 0.7	2.8 ± 0.5	2.6 ± 0.6	3.2 ± 0.8	2.7 ± 0.6	2.7 ± 0.5	2.8 ± 0.7	2.9 ± 0.7
**Demands (Median [Q1-Q3])**	3.1 [2.6–3.5]	2.8 [2.5–3.2]	2.6 [2.2–3.0]	3.3 [2.7–3.8]	2.7 [2.3–3.1]	2.6 [2.2–3.1]	2.8 [2.2–3.5]	2.9 [2.4–3.4]
**Manager’s Support (Mean ± SD)**	2.4 ± 0.8	2.9 ± 0.7	2.1 ± 0.7	3.5 ± 0.9	2.8 ± 0.9	3.6 ± 0.9	2.9 ± 0.9	2.8 ± 1.0
**Manager’s Support (Median [Q1-Q3])**	2.4 [1.8- 3.2]	2.9 [2.6-3.2]	2.1 [1.6-2.6]	3.7 [2.8-4.4]	2.8 [2.2-3.6]	4.0 [3.0-4.4]	3.3 [2.25-3.4]	2.8 [2.0–3.6]
**Peer Support** **(Mean ± SD)**	3.4 ± 0.8	3.2 ± 0.8	3.3 ± 0.8	3.9 ± 0.6	3.6 ± 0.7	3.9 ± 0.5	3.7 ± 0.8	3.6 ± 0.8
**Peer Support** **(Median [Q1-Q3])**	3.7 [3.0-4.0]	3.5 [2.7-3.9]	3.5 [2.7-4.0]	4.0 [3.7-4.5]	3.7 [3.2-4.0]	4.0 [3.5-4.2]	4.0 [3.6-4.0]	3.8 [3.0–4.0]
**Relationship** **(Mean ± SD)**	2.9 ± 0.8	3.1 ± 0.7	2.4 ± 0.8	3.6 ± 1.0	3.0 ± 0.7	2.9 ± 0.9	3.3 ± 0.8	3.0 ± 0.9
**Relationship (Median [Q1-Q3])**	3.0 [2.5-3.5]	3.0 [2.7-3.9]	2.3 [2.0-3.0]	3.7 [3.2-4.5]	3.0 [2.5-3.7]	2.7 [2.2-3.7]	3.2 [2.7-4.0]	3.0 [2.2–3.8]
**Role** **(Mean ± SD)**	3.4 ± 0.7	3.9 ± 0.7	3.5 ± 0.7	4.1 ± 0.6	4.0 ± 0.6	3.9 ± 0.5	3.8 ± 0.6	3.8 ± 0.7
**Role** **(Median [Q1-Q3])**	3.6 [3.0-4.0]	3.9 [3.6-4.7]	3.6 [3.0-4.0]	4.2 [3.8-4.6]	4.0 [3.6-4.4]	4.0 [3.6-4.4]	3.8 [3.4-4.3]	3.8 [3.4–4.2]

**Table 3:** Kruskal–Wallis test results for differences in HSE-MS IT scores across‌‌ PSP occupations.

Variable	H	df	p	ε² (epsilon-squared)
Change	167.17	6	<0.001	0.25
Control	104.48	6	<0.001	0.15
Demands	70.82	6	<0.001	0.10
Manager Support	178.56	6	<0.001	0.27
Peer Support	60.95	6	<0.001	0.09
Relationships	91.73	6	<0.001	0.13
Role	84.79	6	<0.001	0.12
Total Score	153.15	6	<0.001	0.23

df: degrees of freedom, ns: not significant, q: quartile, sd: standard deviation.

When looking at the differences across occupations (S1 Table), where lower HSE-MS IT subscale scores indicate higher stress, we observed that firefighters and police had the highest change scores (Firefighters: mean = 3.1 ± 1.0, median = 3.3 [2.3–4.0] and Police: mean = 3.1 ± 0.9, median = 3.3 [2.6–3.6]). In contrast, border services officers and correctional workers represented the lowest change scores (Border: mean = 1.8 (±0.7), median = 1.67 [1.3–2.3]; Corrections: mean = 1.8 (±0.7), median = 1.6 [1.3–2.3]). Control scores had means ranging from 2.6 to 3.5, with communicators representing the lowest score, with border services officers and correctional workers close behind (mean = 2.6 (±0.8); median = 2.5 [2.0–3.5]), and police the highest (mean = 3.5 (±0.6); median = 3.8 [3.2–4.0]). Firefighters (mean = 3.2 (±0.8); median = 3.3 [2.7–3.8]) and border services officers (mean = 3.1 (±0.7); median = 3.1 [2.6–3.5]) represented higher scores for the demands subscale. Correctional workers reported significantly lower scores for manager’s support (mean = 2.1 (±0.7), median = 2.1 [1.6-2.6]), while firefighters and police had the highest (Firefighters: mean = 3.5 ± 0.9, median = 3.7 [2.8–4.4] and Police: mean = 3.6 ± 0.9, median = 4.0 [3.0–4.4]). On the peer support subscale, the subscale which measures support and resources from colleagues, firefighters and police scored the highest (Firefighters: mean = 3.9 ± 0.6, median = 4.0 [3.7–4.5] and Police: mean = 3.9 ± 0.5, median = 4.0 [3.5–4.2]) while communicators workers had the lowest (Communicators: mean = 3.2 ± 0.8, median = 3.5 [2.7–3.9]). Interestingly, peer support was consistently scored higher across all occupations, with even lower scores were still relatively high (Total: mean = 3.6 (±0.8), median = 3.8 [3.0-4.0]). The relationship subscale measures the promotion of a positive working environment to avoid conflict and deal with unacceptable behavior. Firefighters (mean = 3.6 (±1.0), median = 3.7 [3.2-4.5]) represented the highest score on this subscale, with correctional workers scoring significantly lower than other groups (mean = 2.4 (±0.8), median = 2.3 [2.0-3.0]). Finally, the role subscale scores were the highest in relation to the other subscales (Total: mean = 3.8 (±0.7), median = 3.8 [3.4-4.2]). Firefighters, paramedics, and police showed particularly high role clarity scores, with border services officers and correctional workers reporting slightly lower but still relatively high scores.

Table 4 displays the K6 screening score (means and medians) across all PSP groups. First, we performed a Shapiro-Wilk test and demonstrated that most subscales did not present a normal distribution with W varying from 0.81 to 0.96 (p < 0.001). We then performed a Kruskal–Wallis test to assess whether there was a different distribution of scores across occupations. Significant differences were observed for K6 domains (p < 0.001), with a small effect size (ε² = 0.021–0.063) (Table 5). The Dunn’s post-hoc pairwise comparisons test, with p-values adjusted using the BH method, was performed to demonstrate which groups differ from each other (all p-values are displayed in the S2 Table). Our data demonstrates that correctional workers (mean = 10.8 ± 6.2, median = 10.0 [6.0-16.0]) had a significantly higher total score than firefighters (mean = 6.6 ± 5.2; median = 6.0 [2.0-9.2]), which was the lowest scoring group in our population. Other occupations occupied the middle range, with total K6 scores lower than correctional workers but higher than firefighters.

**Table 4:** Mean and median K6 screening scores.

	Occupations							
	Border Services Officers	Communicators	Correctional Workers	Firefighters	Paramedics	Police	Other	Total
**Respondents (N)**	144	22	151	84	131	88	15	635
**Total Score (Mean ± SD)**	9.1 ± 5.6	8.8 ± 4.5	10.8 ± 6.2	6.6 ± 5.2	8.0 ± 5.2	8.1 ± 3.8	7.8 ± 4.7	8.6 ± 4.5
**Total Score (Median [Q1-Q3])**	9.0 [4.0-13.0]	9.5 [6.0-12.0]	10.0 [6.0-16.0]	6.0 [2.0-9.25]	8.0 [4.0-10.0]	9.0 [6.0-10.0]	8.0 [5.0-10.5]	8.9 [4.0-12.0]

**Table 5:** Kruskal–Wallis test results for differences in K6 scores across PSP occupations.

Variable	H	df	p-value	ε² (epsilon-squared)
Nervous	45.14	6	<0.001	0.062
Hopeless	40.37	6	<0.001	0.055
Restless or fidgety	45.44	6	<0.001	0.063
So depressed that nothing could cheer you up	18.80	6	0.0045	0.021
That everything was an effort	25.47	6	<0.001	0.031
Worthless	22.82	6	<0.001	0.027
K6 Total	32.99	6	<0.001	0.043

df: degrees of freedom, ns: not significant, q: quartile, sd: standard deviation.

When using the K6 as a tool to measure psychological distress, higher scores will indicate greater distress, and the six questions of the tool will suggest what factors might be influencing their physiological distress the most. Overall, our results show that across all the occupations, scores for each question were low to moderate, presenting medians ranging from 0 to 2 on a four-point scale. The themes of the questions with lower scores were hopelessness, depression, and worthlessness, while nervousness, restlessness, and the perception that everything was an effort often received higher scores. When looking at the differences across occupations, we observed that correctional workers often represented higher scores for many questions, including hopelessness (mean = 1.7 ± 1.3, median = 2.0 [0.0–3.0]), nervousness (mean = 2.1 ± 1.0, median = 2.0 [2.0–3.0]), restlessness or fidgety behavior (mean = 2.2 ± 1.1, median = 2.0 [1.0–3.0]), and feelings that everything was an effort (mean = 2.1 ± 1.2, median = 2.0 [1.0–3.0]). Conversely, firefighters had the lowest scores across several questions including hopelessness (mean = 0.8 ± 1.0, median = 0.0 [0.0–1.5]), depression (mean = 0.7 ± 1.0, median = 0.0 [0.0–1.0]), and worthlessness (mean = 0.6 ± 0.9, median = 0.0 [0.0–1.0]). In our sample 22.3% of the PSP workers reported a K6 score higher than or equal to 13, which represents the cut off for potential serious mental illness. Our results demonstrated that 39.1% of correctional workers reported a K6 ≥ 13, followed by border service officers (25.7%), paramedics (19.1%), communicators (18.2%), firefighters (11.9%), and police (9.1%). The prevalence of serious mental illness in our population differed significantly across occupational groups (χ²(6) = 42.1, p < 0.001). A pairwise comparison of proportions was used to show which occupations differed from each other. Correctional workers had a significantly higher prevalence of K6  ≥ 13 compared to firefighters (p < 0.001), paramedics (p = 0.003), and police (p < 0.001). In addition, police had a lower prevalence compared to border services officers (p = 0.018).

Table 6 displays total scores and subscales (means and medians) for the UWES-9 work engagement tool. We first assessed the normality of the distribution of each subscale’s score across occupations, using the Shapiro-Wilk test, and we demonstrated that all the subscales did not present a normal distribution with W ranging from 0.95-0.98 (p < 0.001). To assess whether the scores were differently distributed among occupations, we performed a Kruskal–Wallis test. Significant differences were observed for all UWES-9 subscales (p < 0.001), with a large effect size (ε² = 0.21-0.3) (Table 7). We also performed a Dunn’s post-hoc pairwise comparison test, with p-value adjusted using the BH method to demonstrate which groups differed from each other. All p-values are displayed in the S3 Table. Our results revealed that firefighters (mean = 38.4 ± 8.6, median = 41.0 [34.0 - 45.0]) showed a significantly higher total score than most other occupations, while on the other hand, correctional workers (mean = 21.0 ± 10.7, median = 21.0 [13.0 - 27.0]) and border services officers (mean = 22.6 ± 11.1, median = 22.5 [15.0 – 29.8]) had the lowest work engagement scores.

**Table 6:** UWES-9 mean and median total scores and subscales.

	Occupations							
	Border Services Officers	Communicators	Correctional Workers	Firefighters	Paramedics	Police	Other	Total
**Respondents (N)**	142	22	148	81	125	86	15	619
**Total Score (Out of 54)** **(Mean± SD)**	22.6 ± 11.1	29.3 ± 10.0	21.0 ± 10.7	38.4 ± 8.6	31.3 ± 10.6	35.0 ± 7.3	32.6 ± 8.6	28.3 ± 11.9
**Total Score (Median [Q1-Q3])**	22.5 [15.0 - 29.8]	30.5 [23.8 - 35.0]	21.0 [13.0 - 27.0]	41.0 [34.0-45.0]	33.0 [24.0 - 39.0]	37.0 [32.0 – 40.0]	35.0 [25.0 – 38.0]	29.0 [19.0 - 38.0]
**Total Score** **(Mean ± SD)**	2.5 ± 1.2	3.2 ± 1.1	2.3 ± 1.1	4.2 ± 0.9	3.4 ± 1.1	3.8 ± 0.8	3.6 ± 0.9	3.1 ± 1.3
**Total Score (Median [Q1-Q3])**	2.5 [1.6 - 3.3]	3.3 [2.6 - 3.8]	2.3 [1.4 - 3.0]	4.5 [3.7 - 5.0]	3.6 [2.6 - 3.8]	4.1 [3.5 - 4.4]	3.8 [2.7 - 4.2]	3.2 [2.1 - 4.1]
**Absorption** **(Mean ± SD)**	2.6 ± 1.3	3.2 ± 1.0	2.5 ± 1.3	4.1 ± 0.9	3.4 ± 1.2	3.7 ± 0.8	3.8 ± 1.1	3.1 ± 1.3
**Absorption** **(Median [Q1-Q3])**	2.6 [1.6–3.3]	3.1 [2.4–3.6]	2.6 [1.6–3.3]	4.3 [3.6–5.0]	3.6 [2.6–4.3]	4.0 [3.4–4.3]	3.6 [3.3–4.8]	3.3 [2.3 - 4.0]
**Dedication** **(Mean ± SD)**	2.7 ± 1.5	3.6 ± 1.3	2.2 ± 1.5	4.7 ± 1.1	3.9 ± 1.3	4.2 ± 0.8	4.1 ± 1.1	3.4 ± 1.6
**Dedication** **(Median [Q1-Q3])**	2.6 [1.6–3.6]	3.6 [3.4–4.5]	2.0 [1.0–3.0]	5.0 [4.0–5.6]	4.3 [3.0–5.0]	4.3 [4.0–4.6]	4.3 [3.6–4.8]	3.6 [2.3 - 4.6]
**Vigor** **(Mean ± SD)**	2.1 ± 1.3	2.8 ± 1.3	2.2 ± 1.3	3.9 ± 1.2	3.0 ± 1.3	3.7 ± 1.1	2.9 ± 1.0	2.8 ± 1.4
**Vigor (Median [Q1-Q3])**	2.0 [1.0–3.0]	2.8 [2.3–4.0]	2.0 [1.2–3.3]	4.3 [3.0–5.0]	3.3 [2.0–4.0]	4.0 [3.0–4.6]	3.0 [2.3–3.6]	3.0 [1.6 - 4.0]

**Table 7:** Kruskal–Wallis test results for differences in UWES-9 scores across PSP occupations.

Variable	H	df	p-value	ε² (epsilon-squared)
Vigor	136.13	6	<0.001	0.21
Dedication	190.49	6	<0.001	0.30
Absorption	132.26	6	<0.001	0.21
Total UWES-9	187.79	6	<0.001	0.30

df: degrees of freedom, ns: not significant, q: quartile, sd: standard deviation.

Additionally, we looked at which subscales were driving the differences in total scores across occupations, where higher scores represent higher levels of engagement (S3 Table). In summary, among all PSP groups, UWES-9 subscale scores showed moderate levels of work engagement on the six-point scale, with dedication and absorption often displaying higher scores than vigor. When looking at the first dimension of the UWES-9 tool, absorption, we observed that firefighters had the highest score (mean = 4.1 ± 0.9, median = 4.33 [3.67–5.0]), while border services officers and correctional workers displayed the lowest scores (Border: mean = 2.6 ± 1.3, median = 2.6 [1.6–3.3], Corrections: mean = 2.5 ± 1.3, median = 2.6 [1.6–3.3]). A similar pattern was observed for dedication, were firefighters demonstrated the highest dedication scores (mean = 4.7 ± 1.1, median = 5.0 [4.0–5.6]), followed by police (mean = 4.2 ± 0.8, median = 4.3 [4.0–4.6]), and paramedics (mean = 3.9 ± 1.3, median = 4.3 [3.0–5.0. On the other hand, correctional workers had the dedication lowest scores (mean = 2.2 ± 1.5, median = 2.0 [1.0–3.0]). Finally, vigor had the lowest scores on average of the three subscales, with the highest score coming from firefighters (mean = 3.9 ± 1.2, median = 4.3 [3.0–5.0]) and police (mean = 3.7 ± 1.1, median = 4.0 [3.0–4.6]), while border service officers and correctional workers again showed the lowest scores (Border: mean = 2.1 ± 1.3, median = 2.0 [1.0–3.0]; Corrections: mean = 2.2 ± 1.3, median = 2.0 [1.2–3.3]).

#### Impact of demographic variables on standardized measure scores.

To investigate whether the differences observed across occupations were affected by demographic characteristics that might be unique to each group, we first looked at the distribution of these variables across each tool and subscale. First, we used a Wilcoxon test to check if gender, race, and sexuality presented different HSE-MS IT (work stress), K6 (distress), and UWES-9 (work engagement) scores across each subcategory. All the p-values were BH adjusted. Our results showed minimal gender differences in the HSE-MS IT tool, with only the role score presenting a significant difference (p < 0.05), where women reported slightly higher scores than men. No significant differences were observed in the K6 scores across genders (p > 0.5). Interestingly, strong effects of gender were observed in the work engagement tool (UWES-9). Vigor (p < 0.05), Dedication (p < 0.001), Absorption (p < 0.001) and Total score (p < 0.001), with women reporting higher scores for all of the subscales and total score. Our data did not show any evidence that race is associated with work stress, distress, or work engagement. Additionally, differences by sexuality were observed in several HSE-MS IT subscales, including change, control, and manager support, as well as in overall HSE-MS IT scores (all p < 0.05), where workers identified as LGBTQ+ reported lower scores compared to workers identified as straight. No differences were observed in the K6 scores while only the vigor subscale of the UWES-9 test differed by sexuality (p < 0.05), with workers identified as LGBTQ+ reporting lower scores. However, due to the smaller proportion of races other than “white” and sexuality other than “straight” these results should be interpreted with caution. Finally, to investigate the effects of these variables on the outcomes of each subscale, we performed a multivariable linear regression model to investigate whether differences observed across occupations remained the same after accounting for demographic covariates (gender, race, and sexuality). Our results showed that occupation remained significantly associated with all HSE-MS IT, K6, and UWES-9 outcomes after adjustment (all p-values < 0.001), demonstrating that the differences in work stress, distress, and work engagement observed across occupations are independent of the demographic characteristics of gender, race, and sexuality.

#### Check box and open-text responses.

Of the initial 644 survey participants, 591 responded to the check box and open-text questions. Check boxes allowed respondents to indicate the availability of programs, resources, and supports, their current usage, and intention to use in the future, and results are summarized in frequencies and percentages (Table 8). Overall, the most frequently available employer-funded supports included internal peer support programs (83%), critical incident debriefing (62%), external mental health supports (61%), and extended health benefits (77%) (Table 8). Availability and access patterns varied significantly across professions, with practical support generally showing higher utilization than mental health services. Extended health benefits were most widely offered and accessed service, with 77% of participants reporting availability and 63% reporting actual access. In contrast, on-site mental health services showed low levels of availability and utilization, with only 28% reporting that the service was available, and just 19% accessing it. Internal peer support services were widely available at 83% on average, but accessed by only 38% of those participants, indicating a low rate of use for a highly available support. One paramedic participant described a peer support concern, writing that “*Most people at our organization avoid the peer support at all cost because its run mostly by management and we are afraid of being penalized for discussing what happened and how we feel.”* Critical incident debriefing similarly showed modest access at 36%, despite 62% availability. External mental health supports (without requiring a referral) demonstrated a similar pattern, with 36% access with 61% availability, falling short of the usage of extended health benefits (which could include access to mental health services).

**Table 8:** Available services and intentions for use.

SERVICES	RESPONSES		Border Services Officers		Communicators		Correctional Workers		Firefighters		Paramedics		Police		Other		Total	
	Total (N)		137		21		142		78		119		81		13		591	
**Internal Peer Support Services**	Offered	Accessed	84.67% (116)	43.97% (51)	71.43% (15)	26.67% (4)	78.17% (111)	42.34% (47)	79.49% (62)	33.87% (21)	91.60% (109)	44.04% (48)	87.65% (71)	18.31% (13)	61.54% (8)	62.50% (5)	83.25% (492)	38.41% (189)
		Intend to use		19.83% (23)		26.67% (4)		18.02% (20)		41.94% (26)		21.10% (23)		64.79% (46)		0.00% (0)		28.86% (142)
		Do not intend to use		36.21% (42)		46.67% (7)		39.64% (44)		24.19% (15)		34.86% (38)		16.90% (12)		37.50% (3)		32.72% (161)
	Not offered		8.76% (12)		19.05% (4)		10.56% (15)		12.82% (10)		5.88% (7)		8.64% (7)		38.46% (5)		10.15% (60)	
	Unsure		6.57% (9)		9.52% (2)		11.27% (16)		7.69% (6)		2.52% (3)		3.70% (3)		0.00% (0)		6.60% (39)	
**External Peer Support Services**	Aware	Accessed	12.41% (17)	100.00% (17)	9.52% (2)	100.00% (2)	19.72% (28)	100.00% (28)	23.08% (18)	100.00% (18)	14.29% (17)	100.00% (17)	4.94% (4)	100.00% (4)	38.46% (5)	100.00% (5)	15.40% (91)	100.00% (91)
	Not aware/not accessed		87.59% (120)		90.48% (19)		80.28% (114)		76.92% (60)		85.71% (102)		95.06% (77)		61.54% (8)		84.60% (500)	
**Critical Incident Debriefing**	Offered	Accessed	54.74% (75)	24.00% (18)	61.90% (13)	46.15% (6)	52.11% (74)	39.19% (29)	74.36% (58)	46.55% (27)	59.66% (71)	49.30% (35)	86.42% (70)	20.00% (14)	46.15% (6)	50.00% (3)	62.10% (367)	35.97% (132)
		Intend to use		38.67% (29)		46.15% (6)		31.08% (23)		41.38% (24)		32.39% (23)		72.86% (51)		16.67% (1)		42.78% (157)
		Do not intend to use		37.33% (28)		7.69% (1)		29.73% (22)		12.07% (7)		18.31% (13)		7.14% (5)		33.33% (2)		21.25% (78)
	Not offered		21.17% (29)		38.10% (8)		33.10% (47)		16.67% (13)		30.25% (36)		12.35% (10)		30.77% (4)		24.87% (147)	
	Unsure		24.09% (33)		0.00% (0)		14.79% (21)		8.97% (7)		10.08% (12)		1.23% (1)		23.08% (3)		13.03% (77)	
**External Mental Health Support Service** **(Without a Referral)**	Offered	Accessed	73.72% (101)	45.54% (46)	42.86% (9)	33.33% (3)	42.96% (61)	57.38% (35)	60.26% (47)	23.40% (11)	57.14% (68)	30.88% (21)	77.78% (63)	11.11% (7)	69.23% (9)	55.56% (5)	60.58% (358)	35.75% (128)
		Intend to use		25.74% (26)		33.33% (3)		14.75% (9)		44.68% (21)		38.24% (26)		71.43% (45)		22.22% (2)		36.87% (132)
		Do not intend to use		27.72% (28)		33.33% (3)		27.87% (17)		31.91% (15)		30.88% (21)		17.46% (11)		22.22% (2)		27.09% (97)
	Not offered		10.95% (15)		28.57% (6)		26.76% (38)		21.79% (17)		26.89% (32)		14.81% (12)		15.38% (2)		20.64% (122)	
	Unsure		15.33% (21)		28.57% (6)		30.28% (43)		17.95% (14)		15.97% (19)		7.41% (6)		15.38% (2)		18.78% (111)	
**Internal (On-site) Mental Health Support Services**	Offered	Accessed	32.12% (44)	22.73% (10)	28.57% (6)	66.67% (4)	2.82% (4)	50.00% (2)	38.46% (30)	23.33% (7)	20.17% (24)	8.33% (2)	65.43% (53)	11.32% (6)	23.08% (3)	0.00% (0)	27.75% (164)	18.90% (31)
		Intend to use		31.82% (14)		0.00% (0)		25.00% (1)		60.00% (18)		41.67% (10)		73.58% (39)		33.33% (1)		50.61% (83)
		Do not intend to use		43.18% (19)		33.33% (2)		25.00% (1)		16.67% (5)		50.00% (12)		15.09% (8)		66.67% (2)		29.88% (49)
	Not offered		47.45% (65)		52.38% (11)		80.28% (114)		51.28% (40)		60.50% (72)		27.16% (22)		53.85% (7)		56.01% (331)	
	Unsure		20.44% (28)		19.05% (4)		16.90% (24)		10.26% (8)		19.33% (23)		7.41% (6)		23.08% (3)		16.24% (96)	
**Access to Informational Resources and Training**	Offered	Accessed	70.07% (96)	59.38% (57)	57.14% (12)	41.67% (5)	52.11% (74)	51.35% (38)	62.82% (49)	57.14% (28)	69.75% (83)	54.22% (45)	87.65% (71)	23.94% (17)	76.92% (10)	50.00% (5)	66.84% (395)	49.37% (195)
		Intend to use		21.88% (21)		33.33% (4)		27.03% (20)		28.57% (14)		19.28% (16)		60.56% (43)		30.00% (3)		30.63% (121)
		Do not intend to use		17.71% (17)		25.00% (3)		21.62% (16)		14.29% (7)		26.51% (22)		15.49% (11)		20.00% (2)		19.75% (78)
	Not offered		17.52% (24)		33.33% (7)		30.99% (44)		16.67% (13)		19.33% (23)		4.94% (4)		23.08% (3)		19.97% (118)	
	Unsure		12.41% (17)		9.52% (2)		16.90% (24)		20.51% (16)		10.92% (13)		7.41% (6)		0.00% (0)		13.20% (78)	
**Extended Health Benefits**	Offered	Accessed	83.21% (114)	75.44% (86)	61.90% (13)	53.85% (7)	73.24% (104)	69.23% (72)	57.69% (45)	46.67% (21)	78.99% (94)	71.28% (67)	92.59% (75)	37.33% (28)	84.62% (11)	63.64% (7)	77.16% (456)	63.16% (288)
		Intend to Use		17.54% (20)		30.77% (4)		22.12% (23)		44.44% (20)		24.47% (23)		56.00% (42)		18.18% (2)		29.39% (134)
		Do not intend to use		6.14% (7)		15.38% (2)		8.65% (9)		8.89% (4)		4.26% (4)		6.67% (5)		18.18% (2)		7.24% (33)
	Not offered		8.76% (12)		23.81% (5)		16.90% (24)		25.64% (20)		12.61% (15)		6.17% (5)		15.38% (2)		14.04% (83)	
	Unsure		8.03% (11)		14.29% (3)		9.86% (14)		16.67% (13)		8.40% (10)		1.23% (1)		0.00% (0)		8.80% (52)	
**Flexibility in Work Schedule When Needed**	Offered		43.07% (59)		33.33% (7)		42.25% (60)		55.13% (43)		31.93% (38)		74.07% (60)		61.54% (8)		46.53% (275)	
	Not offered		45.99% (63)		52.38% (11)		47.89% (68)		38.46% (30)		61.34% (73)		23.46% (19)		38.46% (5)		45.52% (269)	
	Unsure		10.95% (15)		14.29% (3)		9.86% (14)		6.41% (5)		6.72% (8)		2.47% (2)		0.00% (0)		7.95% (47)	
**Other Programs and Resources to Support Well-being**	Offered		11.68% (16)		9.52% (2)		7.75% (11)		17.95% (14)		12.61% (15)		55.56% (45)		23.08% (3)		17.94% (106)	
	Not offered		37.96% (52)		47.62% (10)		43.66% (62)		42.31% (33)		47.90% (57)		25.93% (21)		38.46% (5)		40.61% (240)	
	Unsure		50.36% (69)		42.86% (9)		48.59% (69)		39.74% (31)		39.50% (47)		18.52% (15)		38.46% (5)		41.46% (245)	

Notable variations emerged in both service availability and access patterns across public safety occupations (Table 8). Police officers reported high availability of internal peer support services at 88% but showed the lowest access rate at only 18%, contrasting with paramedics who reported the highest availability at 92% and access at 44%. Similarly, for critical incident debriefing, police officers demonstrated 86% availability but only 20% access, while firefighters and paramedics showed lower availability (74% and 60%) but higher utilization rates at 47% and 49% respectively. However, police officers expressed the strongest future intent to use both peer support services (65%) and critical incident debriefing (73%), potentially indicating they recognize the value of these services despite reporting low rates of current use. Work schedule flexibility showed pronounced occupational disparities, with police officers reporting the highest availability at 74% while paramedics reported the lowest at only 32%. Additionally, police reported the highest availability of extended health benefits at 93%, in contrast to paramedics at 79%. These differences in accessibility and usage of supports suggests that police officers may be using different resources and supports than other professions.

The analysis reveals a critical distinction between external and internal mental health services, with participants demonstrating lower access of on-site mental health supports. On-site services were available to substantially fewer participants than external services (28% vs. 61%), and the gap between those with access to on-site services and those using them was even more pronounced than for external services (19% vs. 36%), suggesting that workplace privacy concerns may deter utilization (Table 5). Police exemplified this pattern, with the highest availability of on-site mental health services at 65% but only 11% accessing them.

Following the check box questions, participants were provided with open-text questions soliciting feedback on services. Participants gave varied feedback on their experiences with different types of support. Extended health benefits were the most positively received service, with most respondents expressing satisfaction with their experiences. Peer support services from external organizations also received largely positive feedback, although a minority of open-text respondents endorsed accessing this support. Other employer-provided resources that received largely positive feedback included work schedule flexibility and other programs from employers that fell outside the main categories of the survey and were often unique to the employment setting. Internal peer support services showed less favorable results, with some reporting positive experiences while many gave negative feedback on the service quality; critical incident debriefing received mixed reviews as well. External mental health supports that did not require a referral for access (often provided as general Employee Assistance Programs; EAPs) similarly showed divided satisfaction. Informational resources and training about mental health also received mixed feedback. Overall, participants were most satisfied with extended health benefits where they had flexibility in the professionals they could access and indicated that internal mental health supports would benefit from improvements in service delivery and quality to better address their actual needs and experiences. One firefighter reported EAPs as, *“…garbage, one hour counselling with no follow up,”* while one police participant stated that an internal mental wellness program *“Appeared to be a management exercise, no real value.”*

Beyond their overall experience with services, themes around the availability, quality, and trustworthiness of the services emerged from participant open-text responses. Coverage and availability concerns emerged as the most frequently mentioned issues, particularly regarding extended health benefits with numerous open-text respondents raising these accessibility concerns. Quality concerns were another significant issue, with critical incident debriefing, informational resources and training, and peer support services all being mentioned. A police participant reported not trusting internal services *“to maintain confidentiality and to not suffer repercussions as a result of speaking up,”* and a firefighter participant described management approaches as “*largely performative and virtue signaling on mental health, even using it to get promoted but not actually willing to engage in initiatives that are being requested by employees or that will actually affect change.”* A paramedic noted *“a loss of trust between our internal support team and the front line workers over multiple breaches of privacy,”* and a border services officer characterized informational resources as *“very basic and essentially to ensure the employer ‘checks boxes’ to show they are doing the minimum.”*

### Interviews

There were 15 follow up interview participants from the six main occupational groups, including three border services officers, three correctional workers, three firefighters, and three paramedics, as well as two police officers and one communicator. Due to the low number of communicators in the initial survey (3%, n = 22), it was not possible to recruit more than one communicator to the follow up interviews, despite numerous invitations. Additionally, only two police officers were recruited for interviews, despite a higher number of survey participants compared to communicators, and numerous invitations. Eighty percent of participants were in frontline roles, and 80% reported more than 10 years of experience in their current role. All participants identified their racial identity as white. Just over half identified as women (53.3%), with the remaining participants identifying as men (46.7%). Participants were distributed across Ontario regions, including East (26.7%), Central (33.3%), and West (33.3%), with one participant from the North (6.7%). All participants reported at minimum a college diploma (40.0%), with others reporting undergraduate (33.3%) or graduate-level education (26.7%).

Generally, most participants across fields endorsed most of the employer services inquired about as being offered to them. Internal peer support services, critical incident debriefing, and mental wellness resources or training were reported as available by most participants. Internal mental health services and other employer-funded wellness programs were reported less frequently. All participants in communications, fire, paramedic, and police roles reported having accessed external peer support. Reported access to services varied by sector and employer structure. Border services officers most consistently reported access to internal peer support services, critical incident debriefing, and wellness training. Two border services officers also reported having accessed external peer support. In contrast, participants in municipally governed sectors described more fragmented access. Firefighters reported universal access to critical incident debriefing but did not report access to internal mental health services and reported limited access to broader wellness programs. Police participants reported access to wellness training and internal services, though few reported access to peer support or debriefing. Paramedics reported access to critical debriefing and wellness training, though few reported access to internal peer support or internal mental health services. Correctional workers and the single communicator described uneven access to internal mental health services and critical incident debriefing, despite reporting access to some employer-funded wellness resources.

The most frequently reported workplace concerns included communication of change primarily via email, understaffing, and employer-hosted programs and events being poorly executed or poorly attended by frontline staff. Additional concerns included unclear communication of workplace changes, work demands limiting the ability to review employer communications, role conflict due to competing responsibilities, perceived disconnect between frontline staff and supervisors or management, limited knowledge of workplace resources such as health and safety policies, and insufficient equipment or facilities. Police were least likely to identify concerns about their workplace, while correctional workers, border services officers, and communicators were most likely. Participants described limited access to shared technology as a barrier to their employers’ email-based communication. One correctional worker stated, *“I share one computer with 22 people. And management’s way of sharing information is all via email.”* Staffing shortages were also described as affecting workload and break access. A correctional worker reported, *“because we are so short staffed… we just sometimes we just don’t have the staff available to take breaks,”* while the communicator similarly stated, “*that’s staffing. We were low on staffing.”* A paramedic participant also described workload-related concerns, stating *“we tend to be down staffed… then our workload goes up because we can’t just not see clients.”*

Participants further described concerns related to organizational culture, leadership awareness, and role clarity. Border services officers reported a perceived lack of awareness among senior leadership regarding frontline working conditions, with one participant stating that senior leadership was *“completely unaware… of what’s going on on the front line.”* Some participants described workplace responses to mental health concerns as occurring primarily following serious or high-profile incidents rather than as part of routine practice. Role conflict was also described, particularly among correctional workers, with one participant stating, *“I think my knowledge of an interpretation of my role is very different than what the employer’s interpretation of my role is.”*

Interview data also reflected challenges related to mental health disclosure and support processes. Participants described concerns regarding disclosure of distress within formal workplace systems, particularly where disclosure could trigger organizational processes perceived as isolating. One border services officer described that disclosure of a mental health condition would *“initiate this avalanche of processes behind my back… that would just literally isolate me even more right.”* Correctional workers described concern for colleagues who may not have access to adequate support and noted the absence of consistent follow-up processes, with one participant stating, *“there definitely needs to be more of a formal process, like mandatory mental health check ins.”* Participants also described fatigue associated with shift work and night shifts when discussing mental health supports, with one participant stating, *“people need to understand what it’s like to work shift work and night shift and, you know, going through that fog.”* There was also variability in trust related to peer support services. One firefighter described relying on peers rather than formal critical incident services following difficult calls, stating, “*if I had a really bad call. I wouldn’t be calling my CIS [Critical incident stress] team … I would be calling my FSW [Fire Service Women] peers. Because they better reflect me.”* A police participant similarly described variability in trust related to peer support, stating, *“There’s a bit of a trust thing there [with peer support] sometimes.”* Across roles and sectors, participants described mixed experiences with employer-funded mental wellness supports.

Participants also reported positive workplace factors. Formal harassment or conflict resolution policies were reported by most participants, along with sufficient control over work tasks and clear role or job descriptions. Other reported positive factors included employer-hosted community events, flexibility in work schedules, union or association-hosted community events, and external peer support. Border services officers most frequently reported positive workplace factors, followed by firefighters, paramedics, and police, with correctional workers reporting fewer positive factors. Firefighters described supportive relationships with immediate supervisors, with one participant stating that captains and officers were *“very much a part of the family,”* while also describing a disconnect at higher management levels. Paramedics described workplace policies that allowed supervisors discretion to send staff home following difficult calls without requiring documentation, with one participant stating, *“the employer can send you home paid for the remainder of your shift… and it is used.”*

### Converging survey and interview data

Similarities in themes across data sources were observed during data analysis (Table 9), as well as patterns for occupational groups across the survey’s standardized tools, check box questions and open text comments, and the interview data. For border services officers and correctional workers, their work stress and mental distress was on average higher, while their work engagement was on average lower. In their open-text comments, lower intention for use of internal services was seen, and in the interviews, distrust within the employment environment was a dominant experience. For communicators and paramedics, their work stress and mental distress was closer to average, and their work engagement was average. In their open-text comments, moderate intention for use of internal services was seen, and in the interviews, distrust within the employment environment was a less dominant experience than in comparison to law enforcement careers. For firefighters and police officers, their work stress and mental distress was on average lower, while their work engagement was on average higher. In the open-text comments and interviews of firefighters, average to high intention for use of internal services was seen. Police differed in open-text and in the interviews, with distrust within the employment environment as a dominant experience, with strong concerns about privacy. The link between key themes and data sources is further explored in the following discussion section.

**Table 9:** Converging survey and interview findings.

Key Themes	Survey Standardized Tools	Survey Check Box and Open Text Responses	Semi-structured Interviews
Work Stress	X	X	X
Mental Distress	X	X	X
Work Engagement	X	X	X
Current and future use of internal services		X	X
Current and future use of external services		X	X
Trustworthiness of services		X	X
Unequal access to services		X	X
Communication in the workplace	X	X	X
Positive aspects of the workplace	X	X	X

## Discussion

This study examined Ontario PSP experiences of workplace mental wellness supports using a cross-sectional convergent mixed methods design. Survey findings indicated that participants reported an above average risk of work-related stress, low to moderate level of psychological distress, and low to average work engagement, with significant variation across occupational groups. Qualitative findings from open-text responses and interviews provided critical context for these patterns, illustrating how organizational conditions, access barriers, and perceptions of trust and credibility shape how workplace mental wellness supports are experienced in practice. Taken together, the findings suggest that while mental wellness supports are commonly reported as available, their perceived usefulness and uptake are strongly influenced by organizational and contextual factors rather than by availability alone.

### Responses to standardized measures

On the HSE-MS IT, participants indicated an above average risk of work-related stress, with higher scores indicating lower stress. Overall, firefighters and police scored higher on this measure’s seven subscales, paramedics and communicators tended to score within the mid-range, while correctional workers and border services officers were the lowest-scoring occupations, indicating higher levels of work stress. Areas of work stress that stood out with low scores among the respondents were change, demands, and manager’s support. Scores on control and work relationships fell on average in the mid-range, and scores on peer support and role were higher, indicating these areas as potential points of strength for the respondents within their work environments. Comparing to the following research that also used the HSE-MS IT, our sample scores were consistently lower, indicating higher work-related stressors. A study of 760 Italian municipal workers, which included municipal police, reported mean scores across all seven subscales that were higher than our total sample, indicating greater work stress on average in our sample; however, firefighters and police were the most likely to rate their work conditions comparably to the Italian workers [24]. A British study of 707 employees of a community-based health and social services trust that used the HSE-MS IT to measure work stress produced a similar pattern of results to the Italian study, with our sample scoring lower on average across all subscales [28]. A study of 1038 British correctional workers revealed slightly lower scores in comparison to the Italian municipal and British social services workers, but generally higher scores than our participants other than on peer support, which was similar [29]. A direct comparison to this study’s correctional workers showed our participants scored lower on average on every subscale, often by more than one point [29]. A study of 1,729 British police using the HSE-MS IT revealed similar scores to our total sample [30]; importantly, these scores were noted as falling below the benchmark from a large sample of UK organizations [31]. Police participants in our study scored higher in comparison to the UK police on control, manager’s support, and change by approximately half a point [30]. Generally, in comparison to other public sector employees, our participants scored lower on the HSE-MS IT, indicating higher levels of work-related stress.

On the K6, participants indicated a low to moderate level of psychological distress, with a total mean score of 8.6 (±4.5), ranging from means of 6.6 to 10.8 on the 24-point scale. With scores above 13 on this measure typically indicating serious mental illness, none of the groups met that threshold on average; however, correctional workers exceeded that threshold 39% of the time, at a significantly higher rate than most other occupational groups [32]. Firefighters scored the lowest overall, with paramedics, police, and communicators in the mid-range, and correctional workers and border services officers scoring the highest. General population normative data for the K6 is not commonly reported, but is available from a study of seven European countries, with 7,087 participants, revealing a mean score of 5.2, with means for individual countries falling between 4.1 (the Netherlands) and 6.2 (Poland), with our study population scoring higher on average in comparison [33]. Additionally, a study of 1,709 Japanese employees revealed a mean score of 6.03, with our study population also scoring higher on average [34]. Overall, our participants scored below the threshold for serious mental illness on average, but also scored higher than general population studies from Europe and Asia.

On the UWES-9, participants indicated an average level of work engagement, with total scores ranging from 21.0 to 38.4 on average with a maximum score of 54. Overall, firefighters and police scored higher on this measure’s three six-point subscales (absorption, dedication, vigour), paramedics and communicators tended to score within the mid-range, while correctional workers and border services officers were the lowest-scoring occupations, indicating lower levels of work engagement. Scores were notably high on dedication for firefighters (4.7) and notably low on dedication for correctional workers (2.2) as well as vigour for correctional workers (2.2) and border services officers (2.1). Norms for the UWES-9, based on 12,631 respondents, show a mean total score of 4.05 on the six-point scale (equivalent to 36.45), and on the three six-point subscales, a mean vigour score of 4.18, mean dedication score of 4.28, and mean absorption score of 3.68 [35]. In comparison, our participants scored lower overall on average, with only firefighters and police scoring comparably to the tool norms. On all three subscales, our participants scored lower than the tool norms on average, again with only firefighters and police scoring comparably to the tool norms; notably border services officers and correctional workers scored between one and two points below the norms on the subscales [35]. A study of 235 French police revealed similar scores on the UWES-9 subscales to the police in our study and to the norms for the tool, with French scores higher on average than the border services officers, communicators, correctional workers, and paramedics in our study [36]. Additionally, a study of 1,151 Romanian firefighters scored higher on three subscales than both the tool norms and our participants, including even our firefighter participants [37]. Overall, our participants, excluding firefighters and police, scored below the UWES-9 norms, and below scores from studies of police and firefighters [36,37]. Border services officers and correctional worker participants also scored notably lower than the norms and comparison groups, indicating a significantly lower level of work engagement than other PSP participants.

Overall, across the three standardized measures, a consistent pattern emerged. On average, firefighters and police scored lower on work-related stress, lower on mental distress, and higher on work engagement, paramedics and communicators tended to score within the mid-range on these measures, while correctional workers and border services officers scored with higher levels of work-related stress, higher mental distress, and lower work engagement. This pattern of scoring is similar to responses in a large Canadian PSP survey that measured mental disorder symptoms, with correctional workers more frequently screening positive for a recent mental disorder, communicators and paramedics screening in the mid-range, and firefighters and municipal/provincial police screening less frequently positive for a recent disorder on average [2].

### Positive workplace factors

Although much of the literature on PSP focuses on operational and organizational strain, survey and interview and from the present study reveal several workplace strengths that may act as protective factors across public safety sectors. Most notably, participants widely endorsed the presence of formal harassment and conflict resolution policies, sufficient job control, and clearly defined roles and job descriptions. Additional strengths included flexibility in work schedules, community-building events hosted by employers or associations, and the presence of informal peer support systems. These factors align with broader literature emphasizing the buffering role of autonomy, policy clarity, and collegial support in mitigating the effects of psychological stressors in high-risk occupations [3,4,9]. Supervisor support, co-worker support, and a positive workplace culture have been identified as organizational facilitators of psychological well-being, while clarity in policies and role expectations has been shown to reduce uncertainty and stress [3,4,9].

Firefighter participants reported notably high levels of workplace strengths in comparison to other PSP groups. While firefighters are at high risk for cumulative trauma, their strong peer networks and comparatively lower levels of organizational dysfunction may contribute to lower rates of workers’ compensation claims, as noted in recent findings [6]. These networks are reinforced through targeted peer support initiatives, including programs where active-duty firefighters receive training in mental health and cumulative stress awareness, enabling them to provide informed, confidential support to colleagues. Digital storytelling approaches, such as *Firefighters Helping Firefighters* and *Pocket Peer*, combine personal testimonials with behavioral health education to normalize treatment-seeking and address the psychological impacts of the job [38]. Paramedics similarly noted positive workplace features, including supportive supervisors, recognition for good work, flexibility, and robust conflict resolution pathways. These findings echo existing literature suggesting that paramedics, while highly exposed to trauma, benefit from greater mental health literacy, lower stigma, and more frequent use of support services [6,13].

Border services officers also described a strong sense of control and autonomy in their work, which may be related to the federal standardization of their workplaces. Such features mirror previous findings that policy clarity and role definition can reduce stress and enhance confidence in decision-making [4]. Communicators, though a smaller group in the study, also identified key protective features, including clear job description, informal peer support, and a general sense of control in their workflow, suggesting that even in high-pressure, emotionally demanding roles, clarity, structure, and peer connection can serve as important psychological buffers [3,9]. In contrast, correctional workers reported fewer workplace strengths. Role and policy clarity were noted as positives; however, these were undermined in practice by role conflict, staffing shortages, and interpersonal tensions. Policies on their own are insufficient without consistent and trusted implementation, a limitation also evident in correctional worker accounts [39].

Although protective workplace features are not evenly distributed across PSP sectors, their presence offers insight into possible intervention points. Importantly, these strengths are only effective when paired with authentic leadership engagement and cultural support. As other literature has shown, even well-designed resources become ineffective if workers distrust the systems delivering them or fear stigma for accessing them [39]. Effective protective factors depend not only on the availability of policies and services but also on their consistent, transparent, and equitable application across the organization [3,9]. Maintaining and strengthening these features requires deliberate investment in leadership capacity, ongoing communication, and structured opportunities for employee input to ensure that organizational practices remain relevant and responsive. Building on the strengths identified in this study, particularly peer support, control, and clear work processes and roles, will be essential for creating safer and more sustainable work environments across all public safety sectors.

### Concerning workplace factors

The survey and interview data demonstrate that operational and organizational stressors are key contributors to psychological strain across all PSP groups. While each occupational sector faces distinct demands, common concerns emerged related to staffing, communication, policy implementation, and supervisor relationships. These findings align with previous research highlighting the significance of organizational stressors in shaping PSP mental health outcomes [3,4]. The scale of need reflected in the survey and interview data is consistent with findings that 44.5 percent of Canadian PSP survey participants screened positive for at least one mental disorder, indicating a broad burden of psychological injury within which organizational stressors operate [2].

These patterns map directly onto the organizational domain, where internal structures, colleague and employer relationships, and workplace culture shape psychological risk and recovery [3]. They also align with evidence that staffing shortages heighten strain across different PSP sectors [4,6,40]. Sector-specific concerns revealed important variations. This reflects broader findings that PSP subgroups face different stressors, and these occupational differences have direct implications for intervention design, stigma levels, and RTW outcomes [4,7]. Border services officers and correctional workers were the most likely to report organizational and operational challenges on standardized measures, survey open-text responses, and in interviews. All three correctional worker interview participants described role conflict, staffing shortages, outdated policies, and a lack of confidence in workplace procedures. These concerns closely match existing literature on the correctional environment, which consistently identifies internal structural issues as significant contributors to staff burnout and distress [4]. The prominence of role ambiguity and policy inconsistency in corrections is consistent with work showing that occupationally specific demands and cultures shape exposure profiles and reintegration barriers across PSP groups [4]. Border services officers also reported challenges in communication and staffing. All three interview participants described email-based change announcements as problematic, especially when coupled with high work demands that limited their ability to stay informed. Two of the three also identified inconsistent policy application and poor frontline attendance at employer events. The sole communicator interview participant reported concerns with supervisor support, understaffing, and the overall clarity and accessibility of workplace policies. These concerns are compatible with the broader observation that organizational contributors such as leadership practices and cultural climate influence mental health and help-seeking in PSP settings [3,9].

By contrast, police and fire personnel expressed relatively fewer concerns, while paramedics reported more. One of the two police interview participants mentioned inconsistent policy enforcement and reliance on email communication but did not raise any other significant issues. Among firefighters, two of the three interview participants noted weak employer engagement in fostering workplace relationships and a disconnect between supervisors and frontline staff. Paramedics reported a broader range of challenges, with two interview participants identifying chronic understaffing, limited awareness of available supports, poor execution of employer-led events, and an overreliance on email to communicate organizational change. These differences are reflected in worker’s compensation claim patterns, with firefighters and police more often reporting cumulative trauma and paramedics more often reporting single-event injuries [6,8]. Paramedics also have the highest successful return to work rate in Ontario after psychological injury, which may be linked to greater mental health literacy and lower stigma [6,8].

Survey participants, particularly border services officers, communicators, correctional workers, and paramedics, endorsed above-average levels of work-related stress risks on the HSE-MS IT. Participants also reported mixed views in the survey open-text responses about the effectiveness of employer mental health support efforts, with many indicating limited confidence in the programs offered. These findings are consistent with previous research which identified organizational stressors, such as poor communication, inconsistent supervision, and insufficient structural supports, as critical factors contributing to psychological injury among PSP [3,4,9]. This skepticism mirrors evidence that many workplace initiatives do not sufficiently address structural barriers such as toxic culture, poor leadership, or practical access constraints [3,11]. This finding is also consistent with research linking supervisor support, peer cohesion, and healthy workplace culture to improved mental health and return-to-work trajectories, which underscores why perceived disconnects with management in the interviews are significant [3].

Although operational risks such as trauma exposure are well-established in the PSP literature, multiple studies consistently demonstrate that day-to-day organizational conditions, such as unclear communication, limited engagement with frontline staff, and insufficient leadership support, remain significant sources of distress. Organizational factors are not secondary considerations; they are modifiable elements that exert a substantial influence on program effectiveness, return to work potential, and the reduction of stigma [3,9]. The recurrence of these themes across diverse PSP sectors underscores the urgent need to address internal workplace dynamics in tandem with external operational pressures. When organizational factors are neglected, even the most carefully designed mental health interventions are unlikely to achieve meaningful impact on psychological injury or return to work outcomes. Given the documented system-wide costs associated with psychological injuries, including lost time and disability claims, such organizational problems are likely to have both financial and clinical consequences [7,8]. They may also contribute to return to work challenges in environments perceived as psychologically unsafe, as evidenced in Ontario worker’s compensation analyses [6,8,15]. Lasting change requires that these internal contributors be recognized as central, rather than peripheral, to PSP mental wellness.

### Structural inconsistency of mental wellness supports

Survey responses and interview data indicated that access to workplace mental wellness supports varied substantially across service types and PSP groups. While several supports were commonly reported as available, access to these supports was uneven, and patterns of use differed by program type and occupation. Prior research has identified organizational differences as a key determinant of how resources are implemented and sustained, with decentralized governance models often producing uneven program coverage and variable enforcement [10]. These variations are not only logistical, but systemic, contributing to uneven access to support services, fragmented program implementation, and differential workplace experiences [10]. Federal employees, such as border services officers, typically operate under more centralized governance frameworks, whereas police, firefighters, and paramedics fall under municipal or regional jurisdictions with greater variability in policy enforcement, service delivery, and leadership priorities. Organizational structures have been shown to shape how operational stressors are addressed and how personnel perceive the adequacy of workplace supports [3,4]. These structural discrepancies significantly influence how PSP engage with and benefit from workplace wellness supports, as governance models set the parameters for policy clarity, role definition, and consistency of service delivery. Existing research has identified clear policy frameworks, role clarity, and leadership stability as important conditions for program consistency and credibility [3,4,9,10]. In contrast, PSP in municipally governed sectors described more fragmented access to services. These findings mirror observations that in decentralized systems, leadership priorities and local resource availability often determine which supports are implemented and maintained, resulting in unequal access between departments or regions [10].

The lack of standardization in program access across public safety sectors raises serious concerns about equity and systemic coherence. Decentralized systems without province-wide standards results in inconsistent access to critical supports, especially for those working in under-resourced municipalities or high-turnover departments [10]. The current patchwork approach fails to guarantee equitable care across public safety roles and regions. Federal employees benefit from streamlined policy infrastructures, while municipal personnel may face limited, fluctuating, or inadequately enforced supports. These structural gaps are particularly problematic in high-risk roles, where inconsistent access to peer support, mental health training, and organizational resources can exacerbate operational stressors and hinder recovery from psychological injury [4]. Until mental health interventions are implemented through consistent, sector-wide standards, disparities in service access will persist, further marginalizing high-risk groups and undermining the effectiveness of wellness programming across Canada’s public safety workforce.

### Specific wellness programs

Prior literature has documented widespread implementation of mental wellness programs across public safety sectors, including peer support, psychoeducational initiatives, reactive interventions, and digital training resources. However, evidence regarding the perceived usefulness, credibility, and uptake of these programs remains mixed. Findings from the present study align with this literature, illustrating that the availability of specific wellness programs does not consistently translate into positive or uniform experiences among PSP.

#### Peer support.

Peer support programs are among the most widely implemented workplace interventions, especially in corrections, fire, paramedic, and police services. These programs aim to reduce stigma and improve help-seeking by connecting PSP with trained colleagues. Peer support has also emerged as a frequently studied intervention in the PSP mental health literature. A systematic review identified peer support as the most studied intervention type, with three randomized controlled trials reporting short-term improvements such as reduced stigma and enhanced coping; however, long-term clinical outcomes were rarely assessed, and evidence of sustained mental health improvement was inconsistent, with most studies showing no significant reduction in PTSD symptoms [10]. Peer support has also frequently been embedded within multi-component programs, limiting the availability of standalone evaluations [17]. Findings from the current study align with this mixed evidence base. While internal peer support programs were widely reported as available across PSP groups, survey open-text responses and interview data revealed substantial variability in trust, perceived credibility, and willingness to engage. Participants described concerns related to confidentiality and management involvement, particularly when peer support was perceived as closely linked to organizational hierarchies. These concerns were most pronounced among police and correctional worker participants, reflecting previous research showing that fear of professional or employment-related consequences remains a major barrier to peer support uptake in some public safety settings [41]. Although external peer support programs, often run by professional groups and charities, were mentioned as positive supports by some survey and interview participants, engagement with these services was low overall among participants, making it difficult to identify services that are useful and being regularly engaged with by Ontario PSP.

Recent literature has focused on adapting peer support approaches to better meet the needs of specific public safety groups. Examples include PeerOnCall for paramedics, which aims to increase access to confidential peer support but has faced barriers related to mistrust and workplace norms, and digital storytelling initiatives among firefighters that combine peer narratives with mental health education to normalize treatment-seeking [38]. Police services have similarly expanded peer-led programming, including the introduction of the PeerConnect application in Ontario alongside broader provincial mental health resources [42,43].

Despite these developments, the literature emphasizes that peer support alone is often insufficient to address cumulative psychological stress or produce sustained reductions in PTSD symptoms [38]. The present study complements these findings by demonstrating that peer support is not experienced as uniformly trusted or accessible. Moreover, concerns about confidentiality, particularly the fear that disclosures may affect employment or professional reputation, remain a major barrier to uptake in some public safety groups, such as border services officers, correctional workers, and police, where trust in organizational boundaries is limited [41].

#### Psychoeducational programs.

Findings from the current study indicate that psychoeducational resources and mental health training were commonly reported as available across PSP organizations; however, participants described mixed perceptions of their relevance and usefulness. Survey open-text responses frequently characterized educational materials as generic or insufficiently tailored to frontline realities, while interview participants described difficulties engaging with training that was delivered in formats poorly aligned with operational demands, such as reliance on digital platforms.

These findings align with existing literature on classroom-based psychoeducational programs such as the Road to Mental Readiness (R2MR), which has been widely adopted across public safety organizations. Developed by Canada’s Department of National Defense, R2MR was initially implemented within the Canadian Armed Forces to improve mental health literacy, reduce stigma, and encourage early help-seeking [44]. It is now delivered across police academies, fire services, and correctional institutions, offering psychoeducation on stress and mental health alongside cognitive-behavioural strategies such as goal setting, visualization, cognitive monitoring, and tactical breathing [41]. Despite its widespread uptake, evidence suggests that R2MR primarily influences awareness and help-seeking intentions rather than producing sustained improvements in mental health outcomes [10]. In police services, R2MR is integrated into academy training, showing some promise in raising awareness but with no lasting improvements in stigma, resilience, or workplace engagement [45]. In correctional settings, participants reported increased awareness of distress in themselves, colleagues, and inmates, but studies highlight inconsistent mental health outcomes and difficulty applying strategies in the corrections context [41]. Despite its wide use, R2MR remains limited by its primary focus on individual resilience rather than addressing organizational stressors.

#### Reactive programs.

Findings from the current study indicate that reactive mental health supports, including critical incident debriefing and short-term counseling services, were commonly reported as available across organizations, but were associated with mixed experiences. Survey checkbox and open-text responses highlighted variability in access and satisfaction, alongside a relatively high proportion of concerns related to service quality, follow-up, and trust. Interview participants similarly described uneven experiences with reactive supports across sectors, with some reporting limited perceived usefulness. Participants’ accounts emphasized that reactive supports were often accessed following critical incidents rather than as part of ongoing mental wellness strategies. Several participants described concerns related to the structure and delivery of these services, including perceptions of insufficient follow-up and uncertainty regarding confidentiality when supports were accessed through workplace systems.

These findings are consistent with prior literature examining reactive interventions such as Employee Assistance Programs (EAPs), Critical Incident Stress Debriefing (CISD), and Critical Incident Stress Management (CISM) within PSP settings. EAPs typically provide confidential, short-term counseling through external providers and are used reactively following critical incidents, particularly in correctional settings and among border services officers [14,39]. However, despite their availability, PSP frequently report reluctance to access EAPs due to concerns about confidentiality, stigma, and the superficial nature of the support offered [14,39]. CISD was identified as a frequently studied workplace intervention in a systematic review, with findings showing it was originally structured around a seven-stage debriefing model intended to support psychological recovery following traumatic incidents [10]. However, its effectiveness has been shown to be weak and inconsistent, with one study finding no improvement in mental health outcomes and another reporting increased PTSD symptoms at five-year follow-up [10]; similarly, poor implementation has been observed for CISM [10].These findings raise significant concerns about the quality, delivery, and effectiveness of reactive interventions within PSP workplaces. While correctional and border services organizations often rely on EAPs, CISD, and CISM as primary mental health responses, the limited evidence of their efficacy and low rates of uptake suggest that relying solely on reactive interventions is inadequate for addressing the chronic and cumulative work-related stressors experienced by these workers [10,14,39].

#### Digital training interventions.

Survey data reflected a moderately high level of availability of mental health informational resources and training (on paper/in person and digitally) with a moderate level of uptake. Survey and interview participants indicated some challenges with accessing resources within the workplace, with some fear of being seen looking at print resources, and difficulty privately accessing resources on internal digital platforms in their work environments. While interest was expressed by participants in attending training, the preference was frequently for in person group training, which can be logistically difficult to provide, and has sometimes been replace by online, asynchronous modules that were reported to be less engaging. Online mental health training and digital therapy platforms have become increasingly common, particularly since the COVID-19 pandemic, praised for their flexibility, accessibility, and privacy for PSP who may avoid help due to stigma. Among correctional workers, these tools were initially valued for expanding access and promoting help-seeking through educational content and self-guided cognitive-behavioural strategies [12]. However, both correctional workers and other PSP describe major limitations with these digital approaches; studies demonstrate that PSP have criticized online modules as impersonal and ineffective, often describing them as tasks to complete rather than meaningful training [13]. In a qualitative study of Canadian PSP experiences with mental health training, many participants found the content too general and disconnected from the realities of their roles, making it difficult to engage [13]. Some reported simply clicking through required material with little attention, especially when expected to complete training during off-hours or between calls; communicators noted that screen fatigue from long shifts further reduced their willingness to participate, while firefighters and paramedics viewed the content as bureaucratic and lacking relevance [13]. Many also reported poor timing of training, stating it was either delivered too early in their careers before they could apply it or too late after symptoms had developed. PSP expressed frustration with one-time training sessions and a lack of ongoing learning opportunities. Additional concerns included confidentiality and mistrust, especially when peer involvement was included in digital settings [13]. Overall, while digital tools can improve access and reduce stigma, evidence suggests low engagement, high dropout rates, and poor knowledge retention unless training is tailored, continuous, and delivered by individuals who understand PSP workplace realities [13].

Findings from the current study align with these concerns. Survey respondents reported mixed satisfaction with informational resources and mental health training, alongside frequent concerns regarding quality and relevance. Participants also identified barriers that limited engagement with workplace-delivered information, including reliance on digital communication and limited capacity to review materials within demanding, shift-based work environments. These findings support existing evidence that, while digital tools may improve access in principle, their perceived usefulness is strongly shaped by relevance, delivery context, and integration into PSP work realities.

#### Access to interventions.

Findings from this study revealed high rates of access to wellness interventions, including peer support, debriefing services, and mental health resources. However, both survey responses and interview insights suggested that the availability of these programs does not necessarily translate to effectiveness. Participants across PSP sectors described organizational efforts as misaligned with the realities of their roles, lacking relevance, and often delivered in inaccessible or performative ways. This reflects a wider trend in the literature, where even well-intentioned programs like R2MR and CISD are often implemented without adapting to occupational culture, workload demands, or the specific operational stressors faced by each PSP group [10,13,17,41]. These observations are consistent with critiques that many workplace wellness initiatives are delivered in a checkbox-like manner, failing to foster meaningful engagement or sustained cultural change [13].

Interview data further demonstrated that even when services were technically available, they were often viewed as poorly timed, inauthentic, or disconnected from frontline experiences. Similar concerns emerged in the literature regarding reactive programs such as EAPs and CISM, where inconsistent delivery, weak evidence of long-term benefit, and fears around confidentiality significantly undermined uptake [10,14,17,39]. As such, while interventions are present, they are not embedded in organizational culture in a way that makes them usable or trusted.

Participants across occupational groups expressed that mental health education was often reactive, mirroring patterns identified in program evaluations, where PSP often encounter mental health initiatives only after critical incidents, despite clear evidence that proactive, embedded supports improve long-term resilience and retention [17,41]. This sense of reactive implementation reinforces the idea that wellness programming is more about institutional image than frontline support. These concerns echo findings that workplace interventions often emphasize individual resilience strategies rather than systemic reform [10,17]. Programs such as R2MR, though widely implemented, focus primarily on building individual coping skills while overlooking organizational contributors to distress, including workload, unclear leadership, and role conflict [10].

Peer support, while often more available and frequently accessed than other mental health supports, was also described as inconsistent in quality and scope unless delivered by trained colleagues. Peer-based initiatives, such as the PeerOnCall app for paramedics and digital storytelling platforms for firefighters, have shown potential to reduce stigma and encourage help-seeking; however, concerns about trust and confidentiality continue to limit uptake [10,46,47]. Overall, while digital training tools can improve access and reduce stigma, evidence suggests low engagement, high dropout rates, and poor knowledge retention unless training is tailored, continuous, and delivered by individuals who understand PSP workplace realities [13]. This finding aligns with the literature showing that PSP are less likely to benefit from interventions that do not reflect the emotional demands or structural constraints of their particular roles [13,41].

Overall, the mismatch between the structure of existing wellness programs and the lived experience of PSP highlights the need for a fundamental shift. Interventions that prioritize individual resilience without addressing harmful workplace cultures or leadership inaction will remain limited in impact. Sustained improvement requires leadership accountability, integration of wellness into operational planning, and the use of population-specific program design informed by ongoing evaluation. Without embedding supports into daily practice, programs will continue to be perceived as symbolic rather than substantive, perpetuating the disconnect between organizational policy and frontline reality. Lasting change requires organizations to move beyond checkbox-style programming and toward structural accountability, embedded supports, and context-specific mental health strategies that PSP can access, trust, and use.

### Limitations

The survey was promoted by many public safety unions, organizations, and associations, as well as several employers, and advertised through social media platforms. Since there are no publicly available listings of PSP in Ontario, the research team could not directly advertise to this group. Additionally, there is no definitive count of PSP working in public safety professions in the province, as the organizations that employ them cross many municipalities and jurisdictions. Due to the anonymous nature of the survey, and its posting across multiple platforms, it is possible that duplicate responses are present in the data. Therefore, due to the nature of anonymous survey research, it is not possible to calculate a response rate to the survey; inevitably, some perspectives were not captured, while others may be overrepresented.

Communicators and those falling into the “other” career category participated in the survey and interviews at a lower rate than other occupations, therefore results should be carefully interpreted for this group, as the small size of these groups may affect the comparisons made. Additionally, most correctional workers were provincial while most firefighters and police were municipal. For this reason, the views of communicators, federal correctional workers and police, as well as wildland and volunteer firefighters are potentially underrepresented in this study. The survey and interview participants reflect near gender parity; however, it does not reflect actual workforce composition where some public safety occupations, including firefighting and policing, remain dominated by men. Survey and interview participants also lacked racial and sexual orientation diversity, with a majority of participants identifying as white and straight, and many study participants were mid-career. Although these demographic patterns are common in PSP health research, these limitations reduce the generalizability and inclusiveness of the findings.

## Conclusions

Taken together, the findings from this study demonstrate that while PSP experience shared exposure to psychological risks, their experiences are strongly influenced by the structural, cultural, and occupational contexts in which they work. Each public safety occupational group operates within distinct employment systems and governance structures, which in turn shape access to resources, perceptions of organizational support, and experiences of work-related stress. Study participants reported an above average risk of work-related stress, low to moderate levels of psychological distress, and low to average levels of work engagement, with significant variation across occupational groups. Study findings provided critical context for these patterns, illustrating how organizational factors influence the experiences of workplace mental wellness. While mental wellness supports are commonly reported as available, our study shows that their perceived usefulness and uptake are influenced by organizational and contextual factors rather than by availability alone. This study is the first in Ontario to compare the organizational experiences of PSP across sectors and is exploratory in nature. Future research could seek to theoretically ground these experiences within existing organizational and well-being theories. Additionally, future research should attend to the perspectives of groups present in small numbers in this study, including communicators, gender-, sexuality-, and racially-diverse individuals.

Despite the growing number of mental health and wellness supports in the public safety sector, evidence of lasting impact remains limited [10]. Research shows that without meaningful changes to the workplace itself, these programs are unlikely to succeed alone [9]. Challenges such as poor communication and change management, lack of leadership support and trust, and unequal access to resources reflect deeper structural issues within public safety organizations. To reduce psychological harm and support mental wellness, organizational efforts must address the work conditions that allow harm to persist.

## Supporting information

S1 Table Dunn post-hoc pairwise comparisons for HSE-MS IT subscales with BH adjusted p-values. (DOCX)

S2 Table Dunn post-hoc pairwise comparisons for K6 screening subscales with BH adjusted p-values. (DOCX)

S3 Table Dunn post-hoc pairwise comparisons for UWES-9 subscales with BH adjusted p-values. (DOCX)

## Abbreviations

df: degrees of freedom
ns: not significant
q: quartile
sd: standard deviation
