# Supplementary material for: Ontario public safety personnel experiences of workplace mental wellness supports
Source: PLOS Ment Health. 2026 Jun 8;3(6):e0000558. doi: 10.1371/journal.pmen.0000558 (PMC13245750; doi:10.1371/journal.pmen.0000558)
Supplement: S1 Table — (DOCX) [file pmen.0000558.s001.docx]

**S1 Table. Dunn post-hoc pairwise comparisons for HSE-MS IT subscales with BH adjusted p-values**

| Subscale | Comparison | **Z** | p_value | p_adj | Significance |
| --- | --- | --- | --- | --- | --- |
| Change | Border Services Officers vs Police | 9.35 | 0.0000 | <0.001 | **** |
| Change | Border Services Officers vs Fire | 9.26 | 0.0000 | <0.001 | **** |
| Change | Correctional Workers vs Police | 9.01 | 0.0000 | <0.001 | **** |
| Change | Correctional Workers vs Fire | 8.91 | 0.0000 | <0.001 | **** |
| Change | Paramedic vs Police | 5.37 | 0.0000 | <0.001 | **** |
| Change | Fire vs Paramedic | -5.27 | 0.0000 | <0.001 | **** |
| Change | Border Services Officers vs Paramedic | 4.38 | 0.0000 | <0.001 | **** |
| Change | Correctional Workers vs Paramedic | 3.95 | 0.0001 | <0.001 | *** |
| Change | Communicators vs Police | 3.41 | 0.0007 | 0.0015 | ** |
| Change | Communicators vs Fire | 3.35 | 0.0008 | 0.0017 | ** |
| Change | Other vs Police | 2.93 | 0.0034 | 0.0066 | ** |
| Change | Fire vs Other | -2.88 | 0.0040 | 0.0070 | ** |
| Change | Border Services Officers vs Communicators | 1.98 | 0.0480 | 0.0776 | ns |
| Change | Border Services Officers vs Other | 1.78 | 0.0751 | 0.1126 | ns |
| Change | Communicators vs Correctional Workers | -1.73 | 0.0832 | 0.1165 | ns |
| Change | Correctional Workers vs Other | 1.57 | 0.1172 | 0.1539 | ns |
| Change | Border Services Officers vs Correctional Workers | 0.49 | 0.6220 | 0.7683 | ns |
| Change | Communicators vs Paramedic | 0.32 | 0.7487 | 0.8735 | ns |
| Change | Other vs Paramedic | 0.22 | 0.8289 | 0.9162 | ns |
| Change | Communicators vs Other | 0.05 | 0.9598 | 0.9598 | ns |
| Change | Fire vs Police | 0.09 | 0.9300 | 0.9598 | ns |
| Control | Paramedic vs Police | 7.95 | 0.0000 | <0.001 | **** |
| Control | Correctional Workers vs Police | 7.74 | 0.0000 | <0.001 | **** |
| Control | Fire vs Paramedic | -5.93 | 0.0000 | <0.001 | **** |
| Control | Border Services Officers vs Police | 5.68 | 0.0000 | <0.001 | **** |
| Control | Correctional Workers vs Fire | 5.67 | 0.0000 | <0.001 | **** |
| Control | Communicators vs Police | 4.47 | 0.0000 | <0.001 | **** |
| Control | Border Services Officers vs Fire | 3.62 | 0.0003 | <0.001 | *** |
| Control | Other vs Paramedic | -3.51 | 0.0004 | 0.0012 | ** |
| Control | Communicators vs Fire | 3.30 | 0.0010 | 0.0020 | ** |
| Control | Correctional Workers vs Other | 3.33 | 0.0009 | 0.0020 | ** |
| Control | Communicators vs Other | 2.75 | 0.0060 | 0.0115 | * |
| Control | Border Services Officers vs Paramedic | -2.70 | 0.0068 | 0.0120 | * |
| Control | Border Services Officers vs Correctional Workers | -2.32 | 0.0202 | 0.0324 | * |
| Control | Border Services Officers vs Other | 2.30 | 0.0216 | 0.0324 | * |
| Control | Fire vs Police | 1.85 | 0.0650 | 0.0910 | ns |
| Control | Border Services Officers vs Communicators | -1.30 | 0.1941 | 0.2547 | ns |
| Control | Other vs Police | 0.60 | 0.5494 | 0.6787 | ns |
| Control | Correctional Workers vs Paramedic | -0.46 | 0.6422 | 0.7416 | ns |
| Control | Fire vs Other | 0.42 | 0.6710 | 0.7416 | ns |
| Control | Communicators vs Correctional Workers | 0.12 | 0.9037 | 0.9046 | ns |
| Control | Communicators vs Paramedic | -0.12 | 0.9046 | 0.9046 | ns |
| Demands | Correctional Workers vs Fire | 6.18 | 0.0000 | <0.001 | **** |
| Demands | Border Services Officers vs Correctional Workers | -5.83 | 0.0000 | <0.001 | **** |
| Demands | Fire vs Police | -5.49 | 0.0000 | <0.001 | **** |
| Demands | Fire vs Paramedic | -5.10 | 0.0000 | <0.001 | **** |
| Demands | Border Services Officers vs Police | -5.01 | 0.0000 | <0.001 | **** |
| Demands | Border Services Officers vs Paramedic | -4.59 | 0.0000 | <0.001 | **** |
| Demands | Communicators vs Fire | 2.37 | 0.0179 | 0.0536 | ns |
| Demands | Fire vs Other | -2.22 | 0.0263 | 0.0691 | ns |
| Demands | Border Services Officers vs Communicators | -1.81 | 0.0700 | 0.1634 | ns |
| Demands | Border Services Officers vs Other | -1.72 | 0.0850 | 0.1785 | ns |
| Demands | Border Services Officers vs Fire | 1.11 | 0.2668 | 0.4318 | ns |
| Demands | Communicators vs Correctional Workers | -1.15 | 0.2495 | 0.4318 | ns |
| Demands | Communicators vs Police | -1.10 | 0.2705 | 0.4318 | ns |
| Demands | Correctional Workers vs Paramedic | 1.06 | 0.2879 | 0.4318 | ns |
| Demands | Paramedic vs Police | -0.92 | 0.3586 | 0.5021 | ns |
| Demands | Correctional Workers vs Other | 0.85 | 0.3951 | 0.5076 | ns |
| Demands | Other vs Police | -0.82 | 0.4109 | 0.5076 | ns |
| Demands | Communicators vs Paramedic | -0.59 | 0.5531 | 0.6453 | ns |
| Demands | Other vs Paramedic | -0.37 | 0.7130 | 0.7880 | ns |
| Demands | Communicators vs Other | -0.12 | 0.9051 | 0.9504 | ns |
| Demands | Correctional Workers vs Police | 0.00 | 0.9998 | 0.9998 | ns |
| Manager Support | Correctional Workers vs Police | 10.75 | 0.0000 | <0.001 | **** |
| Manager Support | Correctional Workers vs Fire | 10.00 | 0.0000 | <0.001 | **** |
| Manager Support | Border Services Officers vs Police | 8.32 | 0.0000 | <0.001 | **** |
| Manager Support | Border Services Officers vs Fire | 7.58 | 0.0000 | <0.001 | **** |
| Manager Support | Correctional Workers vs Paramedic | 6.02 | 0.0000 | <0.001 | **** |
| Manager Support | Paramedic vs Police | 5.27 | 0.0000 | <0.001 | **** |
| Manager Support | Fire vs Paramedic | -4.55 | 0.0000 | <0.001 | **** |
| Manager Support | Communicators vs Correctional Workers | -3.56 | 0.0004 | <0.001 | *** |
| Manager Support | Border Services Officers vs Paramedic | 3.32 | 0.0009 | 0.0021 | ** |
| Manager Support | Correctional Workers vs Other | 3.01 | 0.0026 | 0.0055 | ** |
| Manager Support | Border Services Officers vs Correctional Workers | -2.72 | 0.0065 | 0.0124 | * |
| Manager Support | Communicators vs Police | 2.64 | 0.0084 | 0.0146 | * |
| Manager Support | Other vs Police | 2.39 | 0.0169 | 0.0273 | * |
| Manager Support | Communicators vs Fire | 2.22 | 0.0266 | 0.0398 | * |
| Manager Support | Border Services Officers vs Communicators | 2.16 | 0.0305 | 0.0426 | * |
| Manager Support | Fire vs Other | -2.02 | 0.0432 | 0.0567 | ns |
| Manager Support | Border Services Officers vs Other | 1.80 | 0.0718 | 0.0887 | ns |
| Manager Support | Fire vs Police | 0.66 | 0.5077 | 0.5923 | ns |
| Manager Support | Communicators vs Paramedic | -0.42 | 0.6760 | 0.7472 | ns |
| Manager Support | Other vs Paramedic | -0.28 | 0.7758 | 0.8146 | ns |
| Manager Support | Communicators vs Other | -0.06 | 0.9494 | 0.9494 | ns |
| Peer Support | Correctional Workers vs Fire | 6.40 | 0.0000 | <0.001 | **** |
| Peer Support | Correctional Workers vs Police | 5.50 | 0.0000 | <0.001 | **** |
| Peer Support | Border Services Officers vs Fire | 4.61 | 0.0000 | <0.001 | **** |
| Peer Support | Border Services Officers vs Police | 3.72 | 0.0002 | <0.001 | *** |
| Peer Support | Communicators vs Fire | 3.71 | 0.0002 | <0.001 | *** |
| Peer Support | Fire vs Paramedic | -3.56 | 0.0004 | 0.0013 | ** |
| Peer Support | Communicators vs Police | 3.20 | 0.0014 | 0.0041 | ** |
| Peer Support | Correctional Workers vs Paramedic | 3.10 | 0.0019 | 0.0050 | ** |
| Peer Support | Paramedic vs Police | 2.69 | 0.0072 | 0.0169 | * |
| Peer Support | Correctional Workers vs Other | 2.48 | 0.0132 | 0.0277 | * |
| Peer Support | Communicators vs Other | 2.06 | 0.0391 | 0.0746 | ns |
| Peer Support | Border Services Officers vs Correctional Workers | -2.02 | 0.0434 | 0.0760 | ns |
| Peer Support | Communicators vs Paramedic | 1.71 | 0.0864 | 0.1396 | ns |
| Peer Support | Border Services Officers vs Other | 1.58 | 0.1133 | 0.1700 | ns |
| Peer Support | Border Services Officers vs Communicators | -1.14 | 0.2544 | 0.3478 | ns |
| Peer Support | Border Services Officers vs Paramedic | 1.11 | 0.2650 | 0.3478 | ns |
| Peer Support | Other vs Paramedic | -1.07 | 0.2843 | 0.3512 | ns |
| Peer Support | Fire vs Police | -0.80 | 0.4247 | 0.4955 | ns |
| Peer Support | Fire vs Other | -0.76 | 0.4484 | 0.4956 | ns |
| Peer Support | Other vs Police | 0.32 | 0.7525 | 0.7902 | ns |
| Peer Support | Communicators vs Correctional Workers | 0.12 | 0.9081 | 0.9081 | ns |
| Relationships | Correctional Workers vs Fire | 9.28 | 0.0000 | <0.001 | **** |
| Relationships | Border Services Officers vs Fire | 5.16 | 0.0000 | <0.001 | **** |
| Relationships | Correctional Workers vs Paramedic | 5.23 | 0.0000 | <0.001 | **** |
| Relationships | Fire vs Police | -4.87 | 0.0000 | <0.001 | **** |
| Relationships | Border Services Officers vs Correctional Workers | -4.70 | 0.0000 | <0.001 | **** |
| Relationships | Fire vs Paramedic | -4.53 | 0.0000 | <0.001 | **** |
| Relationships | Correctional Workers vs Police | 3.81 | 0.0001 | <0.001 | *** |
| Relationships | Correctional Workers vs Other | 3.44 | 0.0006 | 0.0015 | ** |
| Relationships | Communicators vs Correctional Workers | -3.21 | 0.0013 | 0.0031 | ** |
| Relationships | Communicators vs Fire | 2.14 | 0.0321 | 0.0675 | ns |
| Relationships | Other vs Police | -1.45 | 0.1475 | 0.2816 | ns |
| Relationships | Border Services Officers vs Other | 1.36 | 0.1750 | 0.3063 | ns |
| Relationships | Fire vs Other | -1.25 | 0.2110 | 0.3409 | ns |
| Relationships | Other vs Paramedic | -1.07 | 0.2852 | 0.4278 | ns |
| Relationships | Communicators vs Police | -0.93 | 0.3498 | 0.4898 | ns |
| Relationships | Border Services Officers vs Communicators | 0.82 | 0.4150 | 0.5186 | ns |
| Relationships | Paramedic vs Police | -0.81 | 0.4198 | 0.5186 | ns |
| Relationships | Border Services Officers vs Paramedic | 0.62 | 0.5349 | 0.6240 | ns |
| Relationships | Communicators vs Other | 0.52 | 0.6032 | 0.6579 | ns |
| Relationships | Communicators vs Paramedic | -0.49 | 0.6266 | 0.6579 | ns |
| Relationships | Border Services Officers vs Police | -0.27 | 0.7879 | 0.7879 | ns |
| Role | Correctional Workers vs Fire | 6.59 | 0.0000 | <0.001 | **** |
| Role | Border Services Officers vs Fire | 6.40 | 0.0000 | <0.001 | **** |
| Role | Correctional Workers vs Paramedic | 6.01 | 0.0000 | <0.001 | **** |
| Role | Border Services Officers vs Paramedic | 5.80 | 0.0000 | <0.001 | **** |
| Role | Correctional Workers vs Police | 4.68 | 0.0000 | <0.001 | **** |
| Role | Border Services Officers vs Police | 4.51 | 0.0000 | <0.001 | **** |
| Role | Communicators vs Correctional Workers | -2.74 | 0.0062 | 0.0187 | * |
| Role | Border Services Officers vs Communicators | 2.65 | 0.0081 | 0.0211 | * |
| Role | Correctional Workers vs Other | 1.83 | 0.0680 | 0.1586 | ns |
| Role | Border Services Officers vs Other | 1.75 | 0.0794 | 0.1668 | ns |
| Role | Fire vs Police | -1.70 | 0.0896 | 0.1710 | ns |
| Role | Fire vs Other | -1.48 | 0.1382 | 0.2418 | ns |
| Role | Fire vs Paramedic | -1.23 | 0.2189 | 0.3536 | ns |
| Role | Communicators vs Fire | 1.09 | 0.2777 | 0.4166 | ns |
| Role | Other vs Paramedic | 0.88 | 0.3765 | 0.5271 | ns |
| Role | Paramedic vs Police | -0.63 | 0.5266 | 0.6912 | ns |
| Role | Other vs Police | 0.54 | 0.5885 | 0.7270 | ns |
| Role | Communicators vs Other | -0.44 | 0.6607 | 0.7697 | ns |
| Role | Communicators vs Paramedic | 0.39 | 0.6964 | 0.7697 | ns |
| Role | Border Services Officers vs Correctional Workers | -0.15 | 0.8790 | 0.9230 | ns |
| Role | Communicators vs Police | 0.01 | 0.9907 | 0.9907 | ns |
| Total Score | Correctional Workers vs Fire | 10.36 | 0.0000 | <0.001 | **** |
| Total Score | Correctional Workers vs Police | 9.13 | 0.0000 | <0.001 | **** |
| Total Score | Border Services Officers vs Fire | 7.58 | 0.0000 | <0.001 | **** |
| Total Score | Border Services Officers vs Police | 6.37 | 0.0000 | <0.001 | **** |
| Total Score | Fire vs Paramedic | -5.87 | 0.0000 | <0.001 | **** |
| Total Score | Correctional Workers vs Paramedic | 4.89 | 0.0000 | <0.001 | **** |
| Total Score | Paramedic vs Police | 4.68 | 0.0000 | <0.001 | **** |
| Total Score | Communicators vs Fire | 3.58 | 0.0003 | <0.001 | *** |
| Total Score | Correctional Workers vs Other | 3.39 | 0.0007 | 0.0016 | ** |
| Total Score | Border Services Officers vs Correctional Workers | -3.12 | 0.0018 | 0.0037 | ** |
| Total Score | Communicators vs Police | 2.90 | 0.0038 | 0.0072 | ** |
| Total Score | Communicators vs Correctional Workers | -2.34 | 0.0194 | 0.0340 | * |
| Total Score | Border Services Officers vs Other | 2.00 | 0.0451 | 0.0729 | ns |
| Total Score | Border Services Officers vs Paramedic | 1.82 | 0.0693 | 0.0970 | ns |
| Total Score | Fire vs Other | -1.83 | 0.0674 | 0.0970 | ns |
| Total Score | Other vs Police | 1.22 | 0.2207 | 0.2896 | ns |
| Total Score | Other vs Paramedic | -1.17 | 0.2419 | 0.2989 | ns |
| Total Score | Communicators vs Other | 1.09 | 0.2769 | 0.3061 | ns |
| Total Score | Fire vs Police | -1.09 | 0.2762 | 0.3061 | ns |
| Total Score | Border Services Officers vs Communicators | 0.75 | 0.4561 | 0.4789 | ns |
| Total Score | Communicators vs Paramedic | 0.21 | 0.8362 | 0.8362 | ns |
