# Supplementary material for: Ontario public safety personnel experiences of workplace mental wellness supports
Source: PLOS Ment Health. 2026 Jun 8;3(6):e0000558. doi: 10.1371/journal.pmen.0000558 (PMC13245750; doi:10.1371/journal.pmen.0000558)
Supplement: S2 Table — (DOCX) [file pmen.0000558.s002.docx]

**S2 Table. Dunn post-hoc pairwise comparisons for K6 screening subscales with BH adjusted p-values**

| **Subscale** | **Comparison** | **Z** | **p_value** | **p_adj** | **Significance** |
| --- | --- | --- | --- | --- | --- |
| Hopeless | Correctional Workers vs Fire | -4.78 | 0.0000 | <0.001 | **** |
| Hopeless | Border Services Officers vs Fire | -4.12 | 0.0000 | <0.001 | *** |
| Hopeless | Correctional Workers vs Paramedic | -4.16 | 0.0000 | <0.001 | *** |
| Hopeless | Fire vs Police | 3.86 | 0.0001 | <0.001 | *** |
| Hopeless | Border Services Officers vs Paramedic | -3.40 | 0.0007 | 0.0028 | ** |
| Hopeless | Paramedic vs Police | 3.15 | 0.0016 | 0.0057 | ** |
| Hopeless | Correctional Workers vs Other | -2.34 | 0.0192 | 0.0575 | ns |
| Hopeless | Border Services Officers vs Other | -2.02 | 0.0439 | 0.1023 | ns |
| Hopeless | Other vs Police | 2.04 | 0.0415 | 0.1023 | ns |
| Hopeless | Communicators vs Correctional Workers | 1.62 | 0.1055 | 0.2216 | ns |
| Hopeless | Border Services Officers vs Communicators | -1.23 | 0.2178 | 0.3759 | ns |
| Hopeless | Communicators vs Fire | -1.19 | 0.2327 | 0.3759 | ns |
| Hopeless | Communicators vs Police | 1.28 | 0.2010 | 0.3759 | ns |
| Hopeless | Fire vs Paramedic | 1.12 | 0.2631 | 0.3947 | ns |
| Hopeless | Border Services Officers vs Correctional Workers | 0.75 | 0.4548 | 0.5969 | ns |
| Hopeless | Communicators vs Other | -0.79 | 0.4291 | 0.5969 | ns |
| Hopeless | Communicators vs Paramedic | -0.56 | 0.5750 | 0.6948 | ns |
| Hopeless | Correctional Workers vs Police | -0.48 | 0.6287 | 0.6948 | ns |
| Hopeless | Other vs Paramedic | 0.50 | 0.6189 | 0.6948 | ns |
| Hopeless | Border Services Officers vs Police | 0.17 | 0.8678 | 0.9112 | ns |
| Hopeless | Fire vs Other | 0.08 | 0.9392 | 0.9392 | ns |
| K6 Total | Correctional Workers vs Fire | -5.28 | 0.0000 | <0.001 | **** |
| K6 Total | Correctional Workers vs Paramedic | -3.91 | 0.0001 | <0.001 | *** |
| K6 Total | Border Services Officers vs Fire | -3.49 | 0.0005 | 0.0034 | ** |
| K6 Total | Correctional Workers vs Police | -2.53 | 0.0113 | 0.0540 | ns |
| K6 Total | Fire vs Police | 2.49 | 0.0129 | 0.0540 | ns |
| K6 Total | Border Services Officers vs Correctional Workers | 2.06 | 0.0393 | 0.1178 | ns |
| K6 Total | Communicators vs Fire | -2.12 | 0.0342 | 0.1178 | ns |
| K6 Total | Border Services Officers vs Paramedic | -1.88 | 0.0604 | 0.1587 | ns |
| K6 Total | Fire vs Paramedic | 1.81 | 0.0709 | 0.1655 | ns |
| K6 Total | Correctional Workers vs Other | -1.57 | 0.1175 | 0.2468 | ns |
| K6 Total | Communicators vs Paramedic | -1.11 | 0.2691 | 0.5106 | ns |
| K6 Total | Fire vs Other | 1.05 | 0.2918 | 0.5106 | ns |
| K6 Total | Communicators vs Correctional Workers | 0.93 | 0.3525 | 0.5349 | ns |
| K6 Total | Paramedic vs Police | 0.92 | 0.3566 | 0.5349 | ns |
| K6 Total | Border Services Officers vs Police | -0.74 | 0.4615 | 0.6461 | ns |
| K6 Total | Border Services Officers vs Other | -0.68 | 0.4986 | 0.6517 | ns |
| K6 Total | Communicators vs Other | -0.63 | 0.5275 | 0.6517 | ns |
| K6 Total | Communicators vs Police | -0.54 | 0.5925 | 0.6913 | ns |
| K6 Total | Other vs Police | 0.30 | 0.7637 | 0.8441 | ns |
| K6 Total | Border Services Officers vs Communicators | 0.12 | 0.9029 | 0.9029 | ns |
| K6 Total | Other vs Paramedic | -0.16 | 0.8744 | 0.9029 | ns |
| Nervous | Correctional Workers vs Police | -5.91 | 0.0000 | <0.001 | **** |
| Nervous | Correctional Workers vs Fire | -4.71 | 0.0000 | <0.001 | **** |
| Nervous | Border Services Officers vs Correctional Workers | 4.10 | 0.0000 | <0.001 | *** |
| Nervous | Paramedic vs Police | -3.39 | 0.0007 | 0.0036 | ** |
| Nervous | Correctional Workers vs Paramedic | -2.72 | 0.0065 | 0.0273 | * |
| Nervous | Communicators vs Correctional Workers | 2.66 | 0.0079 | 0.0275 | * |
| Nervous | Border Services Officers vs Police | -2.32 | 0.0202 | 0.0607 | ns |
| Nervous | Fire vs Paramedic | 2.26 | 0.0238 | 0.0624 | ns |
| Nervous | Correctional Workers vs Other | -1.76 | 0.0780 | 0.1820 | ns |
| Nervous | Border Services Officers vs Fire | -1.18 | 0.2363 | 0.4136 | ns |
| Nervous | Border Services Officers vs Paramedic | 1.27 | 0.2038 | 0.4136 | ns |
| Nervous | Communicators vs Paramedic | 1.22 | 0.2222 | 0.4136 | ns |
| Nervous | Other vs Police | -1.13 | 0.2582 | 0.4171 | ns |
| Nervous | Fire vs Police | -0.99 | 0.3203 | 0.4804 | ns |
| Nervous | Communicators vs Police | -0.78 | 0.4343 | 0.6080 | ns |
| Nervous | Border Services Officers vs Communicators | -0.56 | 0.5766 | 0.6739 | ns |
| Nervous | Fire vs Other | 0.59 | 0.5580 | 0.6739 | ns |
| Nervous | Other vs Paramedic | 0.56 | 0.5776 | 0.6739 | ns |
| Nervous | Communicators vs Other | 0.39 | 0.6990 | 0.7726 | ns |
| Nervous | Communicators vs Fire | -0.15 | 0.8846 | 0.9288 | ns |
| Nervous | Border Services Officers vs Other | 0.01 | 0.9952 | 0.9952 | ns |
| Restless or fidgety | Correctional Workers vs Fire | -5.32 | 0.0000 | <0.001 | **** |
| Restless or fidgety | Correctional Workers vs Police | -5.12 | 0.0000 | <0.001 | **** |
| Restless or fidgety | Correctional Workers vs Paramedic | -4.37 | 0.0000 | <0.001 | **** |
| Restless or fidgety | Border Services Officers vs Fire | -3.49 | 0.0005 | 0.0025 | ** |
| Restless or fidgety | Border Services Officers vs Police | -3.28 | 0.0010 | 0.0044 | ** |
| Restless or fidgety | Border Services Officers vs Paramedic | -2.30 | 0.0213 | 0.0745 | ns |
| Restless or fidgety | Border Services Officers vs Correctional Workers | 2.09 | 0.0363 | 0.1088 | ns |
| Restless or fidgety | Communicators vs Correctional Workers | 1.99 | 0.0465 | 0.1222 | ns |
| Restless or fidgety | Correctional Workers vs Other | -1.64 | 0.1018 | 0.2376 | ns |
| Restless or fidgety | Fire vs Paramedic | 1.44 | 0.1509 | 0.3168 | ns |
| Restless or fidgety | Paramedic vs Police | -1.21 | 0.2281 | 0.4354 | ns |
| Restless or fidgety | Communicators vs Fire | -1.13 | 0.2584 | 0.4522 | ns |
| Restless or fidgety | Communicators vs Police | -0.99 | 0.3216 | 0.4824 | ns |
| Restless or fidgety | Fire vs Other | 1.01 | 0.3142 | 0.4824 | ns |
| Restless or fidgety | Border Services Officers vs Communicators | -0.91 | 0.3603 | 0.4923 | ns |
| Restless or fidgety | Other vs Police | -0.89 | 0.3751 | 0.4923 | ns |
| Restless or fidgety | Border Services Officers vs Other | -0.73 | 0.4655 | 0.5750 | ns |
| Restless or fidgety | Communicators vs Paramedic | -0.30 | 0.7628 | 0.8471 | ns |
| Restless or fidgety | Other vs Paramedic | -0.30 | 0.7664 | 0.8471 | ns |
| Restless or fidgety | Fire vs Police | 0.22 | 0.8235 | 0.8646 | ns |
| Restless or fidgety | Communicators vs Other | 0.03 | 0.9728 | 0.9728 | ns |
| So depressed that nothing could cheer you up | Correctional Workers vs Fire | -3.22 | 0.0013 | 0.0237 | * |
| So depressed that nothing could cheer you up | Correctional Workers vs Paramedic | -2.96 | 0.0030 | 0.0237 | * |
| So depressed that nothing could cheer you up | Fire vs Police | 2.93 | 0.0034 | 0.0237 | * |
| So depressed that nothing could cheer you up | Paramedic vs Police | 2.63 | 0.0085 | 0.0449 | * |
| So depressed that nothing could cheer you up | Border Services Officers vs Correctional Workers | 2.00 | 0.0459 | 0.1930 | ns |
| So depressed that nothing could cheer you up | Border Services Officers vs Police | 1.78 | 0.0753 | 0.2634 | ns |
| So depressed that nothing could cheer you up | Communicators vs Fire | -1.64 | 0.1014 | 0.3043 | ns |
| So depressed that nothing could cheer you up | Border Services Officers vs Fire | -1.51 | 0.1318 | 0.3459 | ns |
| So depressed that nothing could cheer you up | Communicators vs Paramedic | -1.33 | 0.1824 | 0.3481 | ns |
| So depressed that nothing could cheer you up | Correctional Workers vs Other | -1.37 | 0.1695 | 0.3481 | ns |
| So depressed that nothing could cheer you up | Other vs Police | 1.36 | 0.1742 | 0.3481 | ns |
| So depressed that nothing could cheer you up | Border Services Officers vs Paramedic | -1.01 | 0.3119 | 0.5384 | ns |
| So depressed that nothing could cheer you up | Communicators vs Other | -0.97 | 0.3333 | 0.5384 | ns |
| So depressed that nothing could cheer you up | Border Services Officers vs Communicators | 0.81 | 0.4188 | 0.6282 | ns |
| So depressed that nothing could cheer you up | Fire vs Paramedic | 0.61 | 0.5434 | 0.7608 | ns |
| So depressed that nothing could cheer you up | Border Services Officers vs Other | -0.51 | 0.6087 | 0.7989 | ns |
| So depressed that nothing could cheer you up | Communicators vs Correctional Workers | 0.21 | 0.8330 | 0.9207 | ns |
| So depressed that nothing could cheer you up | Communicators vs Police | 0.23 | 0.8156 | 0.9207 | ns |
| So depressed that nothing could cheer you up | Fire vs Other | 0.25 | 0.8062 | 0.9207 | ns |
| So depressed that nothing could cheer you up | Correctional Workers vs Police | 0.06 | 0.9558 | 0.9558 | ns |
| So depressed that nothing could cheer you up | Other vs Paramedic | 0.06 | 0.9516 | 0.9558 | ns |
| That everything was an effort | Correctional Workers vs Fire | -4.44 | 0.0000 | <0.001 | *** |
| That everything was an effort | Correctional Workers vs Police | -3.12 | 0.0018 | 0.0190 | * |
| That everything was an effort | Border Services Officers vs Correctional Workers | 2.65 | 0.0080 | 0.0335 | * |
| That everything was an effort | Communicators vs Fire | -2.78 | 0.0054 | 0.0335 | * |
| That everything was an effort | Correctional Workers vs Paramedic | -2.71 | 0.0067 | 0.0335 | * |
| That everything was an effort | Border Services Officers vs Fire | -2.15 | 0.0317 | 0.1109 | ns |
| That everything was an effort | Communicators vs Police | -2.01 | 0.0439 | 0.1181 | ns |
| That everything was an effort | Fire vs Paramedic | 2.00 | 0.0450 | 0.1181 | ns |
| That everything was an effort | Communicators vs Paramedic | -1.67 | 0.0941 | 0.2195 | ns |
| That everything was an effort | Border Services Officers vs Communicators | 1.62 | 0.1049 | 0.2204 | ns |
| That everything was an effort | Fire vs Other | 1.54 | 0.1227 | 0.2343 | ns |
| That everything was an effort | Fire vs Police | 1.22 | 0.2216 | 0.3879 | ns |
| That everything was an effort | Other vs Police | -0.88 | 0.3781 | 0.6107 | ns |
| That everything was an effort | Border Services Officers vs Police | -0.80 | 0.4215 | 0.6322 | ns |
| That everything was an effort | Communicators vs Other | -0.70 | 0.4847 | 0.6468 | ns |
| That everything was an effort | Correctional Workers vs Other | -0.64 | 0.5236 | 0.6468 | ns |
| That everything was an effort | Paramedic vs Police | -0.68 | 0.4949 | 0.6468 | ns |
| That everything was an effort | Other vs Paramedic | -0.56 | 0.5772 | 0.6734 | ns |
| That everything was an effort | Border Services Officers vs Other | 0.51 | 0.6129 | 0.6774 | ns |
| That everything was an effort | Communicators vs Correctional Workers | -0.27 | 0.7884 | 0.8279 | ns |
| That everything was an effort | Border Services Officers vs Paramedic | -0.12 | 0.9035 | 0.9035 | ns |
| Worthless | Correctional Workers vs Fire | -4.10 | 0.0000 | <0.001 | *** |
| Worthless | Border Services Officers vs Fire | -3.29 | 0.0010 | 0.0069 | ** |
| Worthless | Correctional Workers vs Paramedic | -3.30 | 0.0010 | 0.0069 | ** |
| Worthless | Border Services Officers vs Paramedic | -2.39 | 0.0170 | 0.0895 | ns |
| Worthless | Fire vs Police | 2.07 | 0.0387 | 0.1625 | ns |
| Worthless | Correctional Workers vs Police | -1.82 | 0.0695 | 0.2432 | ns |
| Worthless | Communicators vs Fire | -1.43 | 0.1532 | 0.4595 | ns |
| Worthless | Border Services Officers vs Police | -1.02 | 0.3065 | 0.5515 | ns |
| Worthless | Communicators vs Correctional Workers | 0.95 | 0.3414 | 0.5515 | ns |
| Worthless | Correctional Workers vs Other | -1.07 | 0.2842 | 0.5515 | ns |
| Worthless | Fire vs Other | 0.96 | 0.3366 | 0.5515 | ns |
| Worthless | Fire vs Paramedic | 1.17 | 0.2417 | 0.5515 | ns |
| Worthless | Paramedic vs Police | 1.10 | 0.2725 | 0.5515 | ns |
| Worthless | Border Services Officers vs Correctional Workers | 0.89 | 0.3726 | 0.5589 | ns |
| Worthless | Communicators vs Paramedic | -0.77 | 0.4395 | 0.6153 | ns |
| Worthless | Border Services Officers vs Other | -0.68 | 0.4956 | 0.6505 | ns |
| Worthless | Border Services Officers vs Communicators | -0.49 | 0.6242 | 0.7711 | ns |
| Worthless | Other vs Paramedic | -0.39 | 0.6990 | 0.8155 | ns |
| Worthless | Communicators vs Other | -0.22 | 0.8279 | 0.9107 | ns |
| Worthless | Communicators vs Police | -0.11 | 0.9107 | 0.9107 | ns |
| Worthless | Other vs Police | 0.16 | 0.8691 | 0.9107 | ns |
