# Supplementary material for: Ontario public safety personnel experiences of workplace mental wellness supports
Source: PLOS Ment Health. 2026 Jun 8;3(6):e0000558. doi: 10.1371/journal.pmen.0000558 (PMC13245750; doi:10.1371/journal.pmen.0000558)
Supplement: S3 Table — (DOCX) [file pmen.0000558.s003.docx]

**S3 Table. Dunn post-hoc pairwise comparisons for UWES-9 subscales with BH adjusted p-values**

| **Subscale** | **Comparison** | **Z** | **p_value** | **p_adj** | **Significance** |
| --- | --- | --- | --- | --- | --- |
| Absorption | Correctional Workers vs Fire | 9.1 | 0.0000 | <0.001 | **** |
| Absorption | Border Services Officers vs Fire | 8.51 | 0.0000 | <0.001 | **** |
| Absorption | Correctional Workers vs Police | 6.93 | 0.0000 | <0.001 | **** |
| Absorption | Border Services Officers vs Police | 6.35 | 0.0000 | <0.001 | **** |
| Absorption | Correctional Workers vs Paramedic | 5.43 | 0.0000 | <0.001 | **** |
| Absorption | Border Services Officers vs Paramedic | 4.79 | 0.0000 | <0.001 | **** |
| Absorption | Fire vs Paramedic | -4.19 | 0.0000 | <0.001 | **** |
| Absorption | Correctional Workers vs Other | 3.53 | 0.0004 | 0.0011 | ** |
| Absorption | Communicators vs Fire | 3.32 | 0.0009 | 0.0021 | ** |
| Absorption | Border Services Officers vs Other | 3.26 | 0.0011 | 0.0024 | ** |
| Absorption | Communicators vs Correctional Workers | -2.01 | 0.0449 | 0.0680 | ns |
| Absorption | Communicators vs Police | 2.01 | 0.0439 | 0.0680 | ns |
| Absorption | Fire vs Police | -2.05 | 0.0403 | 0.0680 | ns |
| Absorption | Paramedic vs Police | 2 | 0.0453 | 0.0680 | ns |
| Absorption | Border Services Officers vs Communicators | 1.69 | 0.0918 | 0.1286 | ns |
| Absorption | Communicators vs Other | 1.49 | 0.1370 | 0.1798 | ns |
| Absorption | Fire vs Other | -1.07 | 0.2843 | 0.3316 | ns |
| Absorption | Other vs Paramedic | -1.09 | 0.2772 | 0.3316 | ns |
| Absorption | Communicators vs Paramedic | 0.87 | 0.3847 | 0.4252 | ns |
| Absorption | Border Services Officers vs Correctional Workers | -0.61 | 0.5393 | 0.5662 | ns |
| Absorption | Other vs Police | -0.06 | 0.9529 | 0.9529 | ns |
| Dedication | Correctional Workers vs Fire | 11.14 | 0.0000 | <0.001 | **** |
| Dedication | Correctional Workers vs Police | 8.93 | 0.0000 | <0.001 | **** |
| Dedication | Border Services Officers vs Fire | 8.74 | 0.0000 | <0.001 | **** |
| Dedication | Correctional Workers vs Paramedic | 8.63 | 0.0000 | <0.001 | **** |
| Dedication | Border Services Officers vs Police | 6.5 | 0.0000 | <0.001 | **** |
| Dedication | Border Services Officers vs Paramedic | 5.92 | 0.0000 | <0.001 | **** |
| Dedication | Correctional Workers vs Other | 4.15 | 0.0000 | <0.001 | **** |
| Dedication | Communicators vs Correctional Workers | -3.6 | 0.0003 | <0.001 | *** |
| Dedication | Fire vs Paramedic | -3.44 | 0.0006 | 0.0013 | ** |
| Dedication | Border Services Officers vs Other | 2.96 | 0.0031 | 0.0059 | ** |
| Dedication | Communicators vs Fire | 2.98 | 0.0029 | 0.0059 | ** |
| Dedication | Border Services Officers vs Correctional Workers | -2.74 | 0.0061 | 0.0106 | * |
| Dedication | Border Services Officers vs Communicators | 2.19 | 0.0288 | 0.0464 | * |
| Dedication | Fire vs Police | -2.13 | 0.0335 | 0.0502 | ns |
| Dedication | Communicators vs Police | 1.62 | 0.1050 | 0.1470 | ns |
| Dedication | Fire vs Other | -1.47 | 0.1404 | 0.1843 | ns |
| Dedication | Paramedic vs Police | 1.16 | 0.2472 | 0.3053 | ns |
| Dedication | Communicators vs Paramedic | 0.97 | 0.3301 | 0.3851 | ns |
| Dedication | Communicators vs Other | 0.9 | 0.3669 | 0.4056 | ns |
| Dedication | Other vs Paramedic | -0.28 | 0.7783 | 0.7783 | ns |
| Dedication | Other vs Police | 0.3 | 0.7607 | 0.7783 | ns |
| Total Engagement | Correctional Workers vs Fire | 10.72 | 0.0000 | <0.001 | **** |
| Total Engagement | Border Services Officers vs Fire | 9.64 | 0.0000 | <0.001 | **** |
| Total Engagement | Correctional Workers vs Police | 8.86 | 0.0000 | <0.001 | **** |
| Total Engagement | Border Services Officers vs Police | 7.77 | 0.0000 | <0.001 | **** |
| Total Engagement | Correctional Workers vs Paramedic | 7.06 | 0.0000 | <0.001 | **** |
| Total Engagement | Border Services Officers vs Paramedic | 5.84 | 0.0000 | <0.001 | **** |
| Total Engagement | Fire vs Paramedic | -4.38 | 0.0000 | <0.001 | **** |
| Total Engagement | Correctional Workers vs Other | 3.56 | 0.0004 | <0.001 | *** |
| Total Engagement | Communicators vs Fire | 3.37 | 0.0007 | 0.0017 | ** |
| Total Engagement | Border Services Officers vs Other | 3.03 | 0.0024 | 0.0051 | ** |
| Total Engagement | Communicators vs Correctional Workers | -2.94 | 0.0033 | 0.0063 | ** |
| Total Engagement | Paramedic vs Police | 2.46 | 0.0138 | 0.0242 | * |
| Total Engagement | Border Services Officers vs Communicators | 2.32 | 0.0205 | 0.0331 | * |
| Total Engagement | Communicators vs Police | 2.22 | 0.0263 | 0.0395 | * |
| Total Engagement | Fire vs Other | -1.84 | 0.0652 | 0.0913 | ns |
| Total Engagement | Fire vs Police | -1.81 | 0.0703 | 0.0923 | ns |
| Total Engagement | Border Services Officers vs Correctional Workers | -1.19 | 0.2328 | 0.2875 | ns |
| Total Engagement | Communicators vs Other | 0.87 | 0.3819 | 0.4365 | ns |
| Total Engagement | Other vs Police | 0.85 | 0.3950 | 0.4365 | ns |
| Total Engagement | Communicators vs Paramedic | 0.8 | 0.4212 | 0.4422 | ns |
| Total Engagement | Other vs Paramedic | -0.39 | 0.6959 | 0.6959 | ns |
| Vigor | Border Services Officers vs Fire | 8.73 | 0.0000 | <0.001 | **** |
| Vigor | Correctional Workers vs Fire | 8.19 | 0.0000 | <0.001 | **** |
| Vigor | Border Services Officers vs Police | 8.09 | 0.0000 | <0.001 | **** |
| Vigor | Correctional Workers vs Police | 7.54 | 0.0000 | <0.001 | **** |
| Vigor | Border Services Officers vs Paramedic | 5.09 | 0.0000 | <0.001 | **** |
| Vigor | Correctional Workers vs Paramedic | 4.46 | 0.0000 | <0.001 | **** |
| Vigor | Fire vs Paramedic | -4.14 | 0.0000 | <0.001 | *** |
| Vigor | Paramedic vs Police | 3.44 | 0.0006 | 0.0016 | ** |
| Vigor | Communicators vs Fire | 2.81 | 0.0049 | 0.0114 | * |
| Vigor | Fire vs Other | -2.45 | 0.0144 | 0.0302 | * |
| Vigor | Border Services Officers vs Communicators | 2.35 | 0.0187 | 0.0327 | * |
| Vigor | Communicators vs Police | 2.37 | 0.0176 | 0.0327 | * |
| Vigor | Other vs Police | 2.07 | 0.0386 | 0.0623 | ns |
| Vigor | Communicators vs Correctional Workers | -1.99 | 0.0462 | 0.0693 | ns |
| Vigor | Border Services Officers vs Other | 1.94 | 0.0522 | 0.0731 | ns |
| Vigor | Correctional Workers vs Other | 1.64 | 0.1014 | 0.1331 | ns |
| Vigor | Border Services Officers vs Correctional Workers | 0.71 | 0.4789 | 0.5610 | ns |
| Vigor | Fire vs Police | -0.7 | 0.4809 | 0.5610 | ns |
| Vigor | Communicators vs Paramedic | 0.37 | 0.7103 | 0.7570 | ns |
| Vigor | Other vs Paramedic | 0.36 | 0.7210 | 0.7570 | ns |
| Vigor | Communicators vs Other | -0.03 | 0.9721 | 0.9721 | ns |
